# Breastfeeding and subsequent risk of 24 types of maternal cancer: a cohort study of Chinese women

**DOI:** 10.1186/s12937-026-01346-2

**Published:** 2026-06-11

**Authors:** Zhicheng Luo, Wenqiong Wu, Xianzhen Liao, Kekui Xu, Yanhua Zou, Zhaohui Shi, Yingyun Hu, Haifan Xiao, Can Li, Shiyu Cao, Shiyu Wang, Jia Guo, Senmao Zhang, Shipeng Yan

**Affiliations:** 1 Department of Cancer Prevention and Control, Hunan Cancer Hospital/the Affiliated Cancer Hospital of Xiangya School of Medicine, Central South University, 283 Tongzipo Road, Changsha, Hunan 410013 PR China; 2 Department of Epidemiology and Health Statistics, Xiangya School of Public Health, Central South University, Changsha, Hunan PR China; 3 Department of Disease Control, The First Affiliated Hospital of Henan University of CM,, Zhengzhou, PR China; 4 Department of Radiotherapy, Hunan Cancer Hospital/the Affiliated Cancer Hospital of Xiangya School of Medicine, Central South University, Changsha, Hunan PR China

**Keywords:** Breastfeeding, Maternal cancer, Chinese women, Cohort study, Preventive

## Abstract

**Background:**

Existing epidemiological evidence on breastfeeding and cancer risks is inconsistent, and no studies have thoroughly examined the relationship between breastfeeding and each site-specific cancer in China. We aimed to investigate these associations among parous Chinese women.

**Methods:**

A total of 157,454 participants aged 40–75 years from 2012 to 2021 were recruited. Cox proportional hazards model was applied to explore the association between breastfeeding behaviors and cancer risks by hazard ratios (HRs) and corresponding 95% confidence intervals (CIs).

**Results:**

5,542 incident cancer cases were identified with a median follow-up of 4.81 years. The history of breastfeeding was statistically significantly associated with lower risk of breast cancer in the multivariate-adjusted model (HR = 0.78, 95%CI: 0.65–0.94). In addition, the risk of gallbladder cancer increased with increasing months of total breastfeeding (*P*-trend = 0.039), with a 51% increase in risk for each additional 12 months of breastfeeding (HR = 1.51, 95%CI: 1.12–2.02). The risk of breast cancer decreased with increasing months of total breastfeeding (*P*-trend = 0.015), with a 15% reduction in risk for each additional 12 months of breastfeeding (HR = 0.85, 95%CI: 0.76–0.95). The subgroup analyses according to menopausal status shows significant separations for colon-rectum cancer, uterus cancer, oesophagus cancer, and larynx cancer (all *P*-interaction < 0.05). In addition, significant linear associations were suggested concerning gallbladder cancer (*P*-overall = 0.0302, *P*-nonlinear = 0.6558) and breast cancer (*P*-overall = 0.0175, *P*-nonlinear = 0.5544).

**Conclusions:**

This study provides further support for the role of breastfeeding behavior in overall cancer and its 24 site-specific cancers.

**Supplementary Information:**

The online version contains supplementary material available at 10.1186/s12937-026-01346-2.

## Introduction

Cancer represents a major global health challenge, with estimated 14.57% of total deaths and 8.8% of total disability-adjusted life years according to the Global Burden of Disease Study 2021. [1] The burden continues to grow, with projections suggesting 35 million new cases by 2050. [2] Beyond its direct health impacts, cancer creates significant societal and economic consequences, highlighting the urgent need for preventive strategies. While established risk factors like tobacco use and obesity contribute substantially, [1] identifying additional modifiable factors remains crucial for targeted prevention.

Breastfeeding, as a major modifiable reproductive factor, plays a critical role in the health of both mother and infant. [3] Recognized worldwide as the best nutritional source for newborn infants, breastfeeding is extensively practiced, especially in low- and middle-income countries. [4] During motherhood, breastfeeding aids in the reduction of postpartum weight gain through increased caloric expenditure, promotes the release of the hormone oxytocin, facilitates the reduction of uterine size to pre-pregnancy levels, and decreases uterine bleeding. Additionally, the long-term benefits of breastfeeding may contribute to a decreased risk of certain cancers in mothers. [5–8] However, aside from breast cancer, the relationship between breastfeeding and the risk of other maternal cancers remains insufficient and controversial. These inconsistencies may stem from variations in populations, methodologies, tumor locations, histological types and invasiveness. [9–11] Furthermore, there is a paucity of comprehensive studies investigating the relationship between breastfeeding and all cancer subtypes within the same population, which could facilitate the exploration of less prevalent cancers and mitigate publication bias. Therefore, additional research is essential to explore the effect of breastfeeding on the risk of different site-specific cancers, particularly in sufficiently large cohorts.

In this study, we concentrated on two breastfeeding behaviors including history of breastfeeding and total breastfeeding duration. Based on a large-scale prospective cohort study conducted in Hunan province, our main objective was to investigate the association of breastfeeding behavior with subsequent risk of 24 types of maternal cancer. Additionally, to examine the dose-response relationships of the above associations.

## Methods

### Study design and population

We conducted a large-scale prospective cohort study under the framework of the Cancer Screening Program in Urban China (CanSPUC). CanSPUC is a non-profit national cancer screening program co-funded by the Chinese Ministry of Finance and the National Health Commission, launched in October 2012. The details of the CanSPUC have been presented in previously published articles. [12] In brief, the targeted population was residents aged 40–75 years living in the selected communities, and the targeted cancer types were the five most prevalent cancers in urban China, including lung cancer, liver cancer, female breast cancer, gastric cancer and colorectal cancer. All eligible participants who provided written informed consent were interviewed by trained staff to complete a questionnaire that included demographic characteristics, lifestyle factors, personal/family history of diseases, and menstrual and reproductive factors (for women only). Based on an established risk scoring system, participants at high risk for cancer were further invited to undergo clinical examinations for one or more cancer screenings. Finally, all participants who completed the questionnaire were followed up either passively or actively to collect information on their cancer incidence and mortality.

In this study, we used data from CanSPUC conducted in Hunan Province between October 2012 and December 2021, covering a total of five cities (Changsha, Xiangtan, Yueyang, Zhuzhou, and Zhangjiajie). Altogether 319,592 eligible participants were enrolled, of whom 167,308 were women. According to the given exclusion criteria, 157,454 females with valid information were finally included in this study. The flow chart of the included participants in this study is shown in eFigure 1. The study was approved by the Ethics Committee of National Cancer Center/Cancer Hospital, Chinese Academy of Medical Sciences and Peking Union Medical College (number of IRB: CH-PRE-004). The procedure of the study was conducted in accordance with the Declaration of Helsinki, and the informed consent was obtained from all participants. A clinical trial number is not applicable to this study.

### Cohort follow-up and case identification

Follow-up for each participant began at the end of the baseline questionnaire and ended with the first diagnosis of cancer, death, the last documented follow-up contact, or December 31, 2021, whichever came first. The system of our program was linked with Hunan Provincial cancer registration system and death surveillance system to access information on the incidence of cancer. The information was also cross-checked with the records of the provincial medical registration system from the Hunan Provincial Health Commission. All cancer cases were coded according to the International Classification of Diseases (ICD-10) (see eTable 1) and further classified as 24 site-specific cancers. [13] In accordance with methodological recommendations from prior studies, [14–16] cancer subtypes with sufficient case numbers (≥ 200) were included in our primary analysis. Detailed results for rarer cancers (< 200 cases) have been moved to Supplementary Material to preserve full data accessibility and future meta-analyses.

### Exposure assessment

Information on menstrual and reproductive factors was collected by self-reported questionnaire at baseline entry. Menstrual and reproductive factors include age at menarche (< 13, 13, 14, or > 14 years), menopause status (no, yes), surgeries on the reproductive system (no, yes), history of breastfeeding (no, yes), and total breastfeeding duration. Total breastfeeding duration was considered as both continuous and multi-categorical (0, 1–6, 7–12, or > 12 months).

Potential risk factors for cancer were selected based on experience and reports from previous studies. [17, 18] Marital status was classified as married or others (including unmarried, widowed, or divorced). Educational level was classified as low (elementary school or below), medium (junior or high school or equivalent), and high (college or above). Smoking status was defined as current (> 1 cigarette/day for at least 6 months), former (previously smoked and have quit for at least 6 months), and never smoking. Alcohol drinking status was classified as never and ever (current alcohol drinking and former alcohol drinking). Regular exercise (no, yes) was defined as an average of more than 3 times per week for more than 30 min per session. History of hypertension (no, yes) and diabetes (no, yes) referred to previous clinical diagnoses of hypertension and diabetes, respectively. Family history of cancer (no, yes) was defined as previous clinical diagnosis of cancer within participants’ three generations. Body mass index (BMI) was calculated as weight in kilograms divided by the square of height in meters, and was further classified into four categories according to the Chinese standard: underweight (< 18.5 kg/m^2^), normal (18.5–23.9 kg/m^2^), overweight (24.0–27.9 kg/m^2^) or obese (≥ 28.0 kg/m^2^).

### Statistical analysis

Descriptive analyses were described as counts and proportions for categorical variables, and mean ± standard deviation (SD) for continuous variables. Chi-square tests and t-tests were applied to compare the differences between groups. The hazard ratios (HRs) and 95% confidence intervals (CIs) of developing cancer in relationship to baseline breastfeeding behavior were evaluated using Cox proportional hazards models. In addition, the associations for breastfeeding history (yes/no) and total breastfeeding duration (continuous and categorical) were evaluated in separate Cox proportional hazards models, because including both variables simultaneously would cause collinearity due to their deterministic relationship. Unadjusted model did not include any covariates; Age-adjusted model only adjusted for age at enrollment (continuous); Multivariate-adjusted model further adjusted for educational level (low, medium, high), marital status (married, others), smoking status (never, current, ever), alcohol drinking status (never, ever), regular exercise (no, yes), BMI (underweight, normal, overweight, obese), family history of cancer (no, yes), hypertension (no, yes), diabetes (no, yes), and age at menarche (< 13, 13, 14, > 14 years), and menopause status (no, yes).

For the continuous variable (total breastfeeding duration), both the dose-response relationship and tests for non-linear associations were performed using restricted cubic spline models with three knots. [19] To examine effect modification, we conducted subgroup analyses for menopausal status. The associations were further investigated in multivariable models among premenopausal and postmenopausal women. Statistical differences between subgroups were investigated by including an interaction term of breastfeeding behaviors and menopausal status in the model. [20].

A series of sensitivity analyses were additionally conducted. Firstly, to mitigate false positives resulting from multiple comparisons, we implemented two complementary approaches: (i) reporting 99%CIs (in addition to conventional 95%CIs) to apply more stringent significance thresholds; (ii) adjusting *P*-values using the Benjamini-Hochberg false discovery rate correction. Secondly, given that surgery on the reproductive system was a significant determinant of cancer incidence and was only collected from 2012 to 2019, we performed additional sensitivity analyses restricted to the 2012–2019 subset with further adjustment for surgeries on the reproductive system in the multivariate-adjusted model. Finally, to evaluate the robustness of the main findings, we included nulliparous female participants into analysis, and took the history of breastfeeding and total breastfeeding duration as none/zero.

Tests in this study were all two-sided and *P* < 0.05 was considered statistically significant. All statistical analyses were conducted using the R software, version 4.2.2.

## Results

During a median follow-up of 4.81 years, 5,542 of 157,454 women were diagnosed with cancer in the cohort, with an incidence density of 82.67/10^5^ person-years. Table 1 presents the baseline characteristics of the study population according to cancer status. Participants with a history of breastfeeding tended to be older, less educated, and more likely to be never-smokers and never-drinkers. They also had lower prevalence of family cancer history, but higher rates of hypertension and diabetes. Additionally, these participants exhibited later menarche age, were more frequently postmenopausal, had fewer reproductive system surgeries, and reported more physical activity.

**Table 1:** Distributions of selected variables of the participants at baseline (2012–2021)

Variables	Person-years	Non-breastfeeding (*n* = 15641)	Breastfeeding (*n* = 141813)	*P* value
Age at enrollment (year, Mean ± SD)	760401.65	56.08 (9.05)	57.09 (9.76)	< 0.001
Educational level (n, %)				
Low	161136.02	2683 (17.2)	33,763 (23.8)	< 0.001
Medium	509830.33	11,238 (71.8)	93,503 (65.9)	
High	89435.30	1720 (11.0)	14,547 (10.3)	
Marital status (n, %)				
Married	734382.37	15,124 (96.7)	136,718 (96.4)	0.069
Others	26019.28	517 (3.3)	5095 (3.6)	
Smoking status (n, %)				
Never	722837.85	13,845 (88.6)	135,651 (95.7)	< 0.001
Current	32440.26	1681 (10.8)	5421 (3.8)	
Ever	3874.36	107 (0.7)	604 (0.4)	
Alcohol drinking status (n, %)				
Never	706226.18	13,314 (85.1)	132,868 (93.7)	< 0.001
Ever	54175.46	2327 (14.9)	8945 (6.3)	
BMI (kg/m^2^, Mean ± SD)		22.99 (2.65)	22.96 (2.65)	0.250
Underweight (< 18.5)	19472.72	383 (2.5)	3610 (2.5)	0.507
Normal (18.5–23.9)	502670.36	10,473 (67.0)	94,229 (66.5)	
Overweight (24.0-27.9)	207692.96	4161 (26.6)	38,132 (26.9)	
Obese (≥ 28.0)	29834.26	607 (3.9)	5721 (4.0)	
Family history of cancer (n, %)				
No	578010.71	8200 (52.4)	109,190 (77.0)	< 0.001
Yes	182390.93	7441 (47.6)	32,623 (23.0)	
Hypertension (n, %)				
No	695834.69	14,477 (92.6)	129,018 (91.0)	< 0.001
Yes	64566.95	1164 (7.4)	12,795 (9.0)	
Diabetes (n, %)				
No	741354.57	15,358 (98.2)	138,647 (97.8)	0.001
Yes	19047.07	283 (1.8)	3166 (2.2)	
Age at menarche (year, Mean ± SD)		13.70 (1.62)	13.90 (1.43)	< 0.001
< 13	96891.13	3516 (22.5)	21,085 (14.9)	< 0.001
13	184815.58	3373 (21.6)	35,188 (24.8)	
14	243614.91	4387 (28.0)	44,095 (31.1)	
> 14	235080.02	4365 (27.9)	41,445 (29.2)	
Menopause status (n, %)				
No	267642.67	5511 (35.2)	44,822 (31.6)	< 0.001
Yes	492758.98	10,130 (64.8)	96,991 (68.4)	
Surgeries on the reproductive system^*^ (n, %)				
No	655753.62	8758 (82.8)	100,845 (91.7)	< 0.001
Yes	71180.30	1823 (17.2)	9088 (8.3)	
Exercise				
No	477067.99	11,190 (71.5)	87,339 (61.6)	< 0.001
Yes	283333.66	4451 (28.5)	54,474 (38.4)	

*Abbreviations*: *SD* Standard deviation, *BMI* Body mass index

^*^The information on female reproductive system surgery was only collected between 2012 and 2019

Table 2 and eTable 2 show the associations of breastfeeding history with cancer incidence in three models (unadjusted model, age-adjusted model, and multivariate-adjusted model). The history of breastfeeding was statistically significantly associated with lower risk of breast cancer in the three models (all *P* < 0.05). No statistically significant associations were observed between history of breastfeeding and other site-specific cancers.

**Table 2:** Risk of developing overall cancer and its main site-specific cancers in relationship to history of breastfeeding

History of breastfeeding	Cases (*n* = 5542)	Unadjusted model	Age-adjusted model	Multivariate-adjusted model
		HR (95%CI)	HR (95%CI)	HR (95%CI)
Colon-rectum (C18-21)				
No	47	Ref.	Ref.	Ref.
Yes	527	1.04 (0.77–1.41)	0.99 (0.73–1.34)	1.04 (0.77–1.40)
Lung (C33-34)				
No	65	Ref.	Ref.	Ref.
Yes	739	1.05 (0.81–1.35)	0.99 (0.77–1.28)	1.07 (0.83–1.39)
Breast (C50)				
No	128	Ref.	Ref.	Ref.
Yes	1000	0.73 (0.61–0.88)	0.73 (0.61–0.88)	0.78 (0.65–0.94)
Cervix (C53)				
No	32	Ref.	Ref.	Ref.
Yes	375	1.10 (0.77–1.58)	1.11 (0.77–1.59)	1.17 (0.81–1.69)
Uterus (C54-55)				
No	18	Ref.	Ref.	Ref.
Yes	222	1.14 (0.71–1.84)	1.14 (0.70–1.84)	1.14 (0.70–1.85)
Brain (C70-72)				
No	20	Ref.	Ref.	Ref.
Yes	251	1.15 (0.73–1.81)	1.10 (0.70–1.74)	1.14 (0.72–1.80)
Thyroid (C73)				
No	47	Ref.	Ref.	Ref.
Yes	425	0.81 (0.60–1.10)	0.83 (0.62–1.12)	0.92 (0.68–1.26)
All others (NA)				
No	29	Ref.	Ref.	Ref.
Yes	363	1.17 (0.80–1.71)	1.14 (0.78–1.66)	1.19 (0.81–1.76)
All sites (C00-97, D32-33, D42-43, D45-47)				
No	487	Ref.	Ref.	Ref.
Yes	5055	0.96 (0.87–1.05)	0.94 (0.85–1.03)	0.99 (0.90–1.09)

Unadjusted model: no covariates were controlled for

Age-adjusted model: only age at enrollment (continuous) was controlled for

Multivariate-adjusted model: age-adjusted model + educational level (low, medium, high), marital status (married, others), smoking status (never, current, ever), alcohol drinking status (never, ever), regular exercise (no, yes), BMI (underweight, normal, overweight, obese), family history of cancer (no, yes), hypertension (no, yes), diabetes (no, yes), age at menarche (< 13, 13, 14, > 14 years), and menopause status (no, yes)

*Abbreviations*: *HR* Hazard ratio, *CI* Confidence interval

Table 3 and eTable 3 present the associations of total breastfeeding duration (continuous and multi-categorical) with cancer incidence in three models. In the multivariate-adjusted model, the risk of gallbladder cancer increased with increasing months of total breastfeeding (*P*-trend = 0.039), with a 51% increase in risk for each additional 12 months of breastfeeding (HR = 1.51, 95%CI: 1.12–2.02). The risk of breast cancer decreased with increasing months of total breastfeeding (*P*-trend = 0.015), with a 15% reduction in risk for each additional 12 months of breastfeeding (HR = 0.85, 95%CI: 0.76–0.95). With respect to thyroid cancer, the risk decreased by 17% with each additional 12 months of breastfeeding (HR = 0.83, 95% CI: 0.69–0.99), although the trend was not statistically significant (*P*-trend = 0.107) in the multivariate-adjusted model. Similarly, the risk of overall cancer incidence decreased by 5% with each additional 12 months of breastfeeding (HR = 0.95, 95% CI: 0.90–0.99), although the trend was not statistically significant (*P*-trend = 0.225).

**Table 3:** Risk of developing overall cancer and its main site-specific cancers in relationship to baseline total breastfeeding duration

Site-specific cancers	Total breastfeeding duration	Cases (*n* = 5542)	Unadjusted model	Age-adjusted model	Multivariate-adjusted model
			HR (95%CI)	HR (95%CI)	HR (95%CI)
Colon-rectum (C18-21)	Continuous (per 12 months)	574	0.97 (0.84–1.12)	0.84 (0.73–0.98)	0.87 (0.75–1.01)
	Multi-categorical (month)				
	0	47	Ref.	Ref.	Ref.
	1–6	79	1.22 (0.85–1.75)	1.25 (0.87–1.80)	1.25 (0.87–1.80)
	7–12	335	1.01 (0.75–1.37)	0.99 (0.73–1.35)	1.03 (0.76–1.41)
	> 12	113	1.03 (0.73–1.45)	0.86 (0.61–1.21)	0.92 (0.65–1.30)
	*P* for trend		0.647	0.079	0.216
Lung (C33-34)	Continuous (per 12 months)	804	1.07 (0.95–1.20)	0.93 (0.83–1.05)	0.97 (0.86–1.09)
	Multi-categorical (month)				
	0	65	Ref.	Ref.	Ref.
	1–6	97	1.07 (0.78–1.47)	1.09 (0.80–1.50)	1.13 (0.82–1.54)
	7–12	465	1.01 (0.78–1.31)	0.99 (0.76–1.28)	1.07 (0.82–1.39)
	> 12	177	1.15 (0.87–1.53)	0.96 (0.73–1.28)	1.06 (0.79–1.42)
	*P* for trend		0.381	0.517	0.915
Breast (C50)	Continuous (per 12 months)	1128	0.83 (0.74–0.92)	0.82 (0.73–0.92)	0.85 (0.76–0.95)
	Multi-categorical (month)				
	0	128	Ref.	Ref.	Ref.
	1–6	139	0.79 (0.62–1.01)	0.79 (0.62–1.01)	0.82 (0.64–1.04)
	7–12	654	0.73 (0.60–0.88)	0.73 (0.60–0.88)	0.78 (0.64–0.94)
	> 12	207	0.70 (0.56–0.87)	0.69 (0.55–0.86)	0.75 (0.60–0.94)
	*P* for trend		0.002	0.001	0.015
Cervix (C53)	Continuous (per 12 months)	407	1.05 (0.89–1.24)	1.07 (0.90–1.27)	1.09 (0.91–1.30)
	Multi-categorical (month)				
	0	32	Ref.	Ref.	Ref.
	1–6	42	0.96 (0.61–1.52)	0.96 (0.60–1.52)	1.01 (0.64–1.60)
	7–12	246	1.10 (0.76–1.59)	1.10 (0.76–1.59)	1.17 (0.81–1.71)
	> 12	87	1.19 (0.79–1.78)	1.22 (0.81–1.83)	1.30 (0.86–1.96)
	*P* for trend		0.253	0.197	0.130
Uterus (C54-55)	Continuous (per 12 months)	240	1.11 (0.90–1.38)	1.11 (0.90–1.38)	1.13 (0.90–1.41)
	Multi-categorical (month)				
	0	18	Ref.	Ref.	Ref.
	1–6	18	0.72 (0.37–1.38)	0.72 (0.37–1.38)	0.73 (0.38–1.40)
	7–12	156	1.22 (0.75–1.99)	1.22 (0.75–1.99)	1.23 (0.75–2.01)
	> 12	48	1.14 (0.66–1.96)	1.14 (0.66–1.96)	1.16 (0.67–2.01)
	*P* for trend		0.218	0.222	0.215
Brain (C70-72)	Continuous (per 12 months)	271	1.14 (0.93–1.39)	1.02 (0.84–1.25)	1.00 (0.82–1.23)
	Multi-categorical (month)				
	0	20	Ref.	Ref.	Ref.
	1–6	29	1.03 (0.58–1.82)	1.05 (0.59–1.85)	1.08 (0.61–1.92)
	7–12	163	1.14 (0.71–1.81)	1.12 (0.70–1.78)	1.16 (0.73–1.86)
	> 12	59	1.26 (0.76–2.09)	1.09 (0.66–1.81)	1.09 (0.65–1.84)
	*P* for trend		0.287	0.712	0.743
Thyroid (C73)	Continuous (per 12 months)	472	0.72 (0.61–0.86)	0.77 (0.65–0.93)	0.83 (0.69–0.99)
	Multi-categorical (month)				
	0	47	Ref.	Ref.	Ref.
	1–6	69	1.03 (0.71–1.49)	1.02 (0.70–1.47)	1.07 (0.73–1.55)
	7–12	286	0.84 (0.61–1.14)	0.84 (0.62–1.14)	0.93 (0.68–1.28)
	> 12	70	0.62 (0.43–0.90)	0.68 (0.47–0.99)	0.78 (0.53–1.14)
	*P* for trend		0.002	0.014	0.107
All others (NA)	Continuous (per 12 months)	392	1.15 (0.97–1.35)	1.04 (0.89–1.23)	1.05 (0.89–1.24)
	Multi-categorical (month)				
	0	29	Ref.	Ref.	Ref.
	1–6	37	0.93 (0.57–1.52)	0.95 (0.58–1.55)	0.99 (0.60–1.62)
	7–12	243	1.20 (0.82–1.76)	1.19 (0.81–1.74)	1.25 (0.84–1.86)
	> 12	83	1.24 (0.81–1.89)	1.10 (0.72–1.67)	1.15 (0.74–1.77)
	*P* for trend		0.146	0.462	0.371
All sites (C00-97, D32-33, D42-43, D45-47)	Continuous (per 12 months)	5542	0.99 (0.95–1.04)	0.93 (0.88–0.97)	0.95 (0.90–0.99)
	Multi-categorical (month)				
	0	487	Ref.	Ref.	Ref.
	1–6	666	0.98 (0.87–1.11)	0.99 (0.88–1.12)	1.02 (0.91–1.15)
	7–12	3275	0.95 (0.86–1.04)	0.94 (0.85–1.03)	0.99 (0.90–1.10)
	> 12	1114	0.98 (0.88–1.09)	0.89 (0.80–0.99)	0.95 (0.85–1.06)
	*P* for trend		0.543	0.012	0.225

Unadjusted model: no covariates were controlled for

Age-adjusted model: only age at enrollment (continuous) was controlled for

Multivariate-adjusted model: Age-adjusted model + educational level (low, medium, high), marital status (married, others), smoking status (never, current, ever), alcohol drinking status (never, ever), regular exercise (no, yes), BMI (underweight, normal, overweight, obese), family history of cancer (no, yes), hypertension (no, yes), diabetes (no, yes), age at menarche (< 13, 13, 14, > 14 years), and menopause status (no, yes)

*Abbreviations*: *HR* Hazard ratio, *CI* Confidence interval

**Fig. 1:**
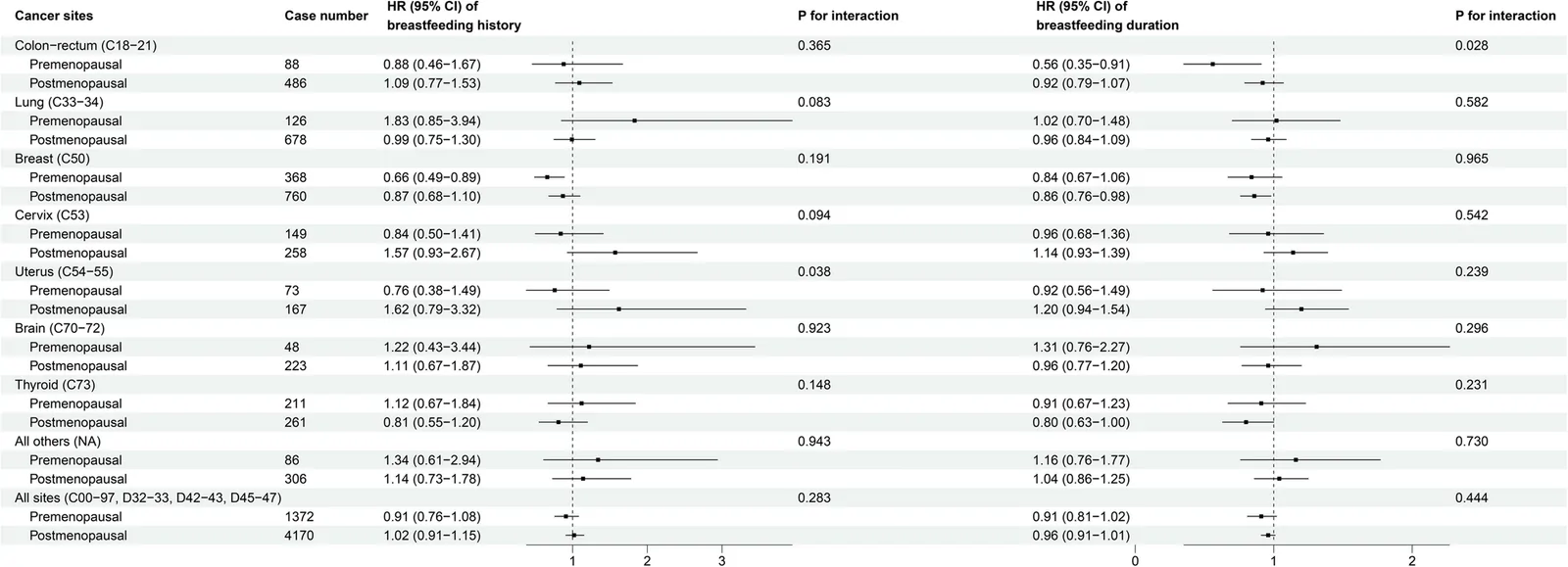
Risk of developing overall cancer and its main site-specific cancers in relationship to breastfeeding behavior stratified by menopausal status

Figure 1 and eTable 4 display the associations between breastfeeding behavior and cancer incidence stratified by menopausal status. The inverse associations of total breastfeeding duration with colon-rectum cancer (0.56 [0.35–0.91] vs. 0.92 [0.79–1.07], *P*-interaction = 0.028), and history of breastfeeding with uterus cancer (0.76 [0.38–1.49] vs. 1.62 [0.79–3.32]), *P*-interaction = 0.038), were more pronounced in premenopausal women compared with those in postmenopausal women. With respect to oesophagus cancer (6.01 [1.46–24.70] vs. 0.79 [0.38–1.65], *P*-interaction = 0.037) and larynx cancer (12.62 [2.69–59.25] vs. 0.37 [0.11–1.22], *P*-interaction = 0.002), stronger positive associations with total breastfeeding duration were observed in premenopausal women than those in postmenopausal women. Notably, the results on oesophagus cancer and larynx cancer should be treated cautiously because of the extremely low number of cancer cases.

**Fig. 2:**
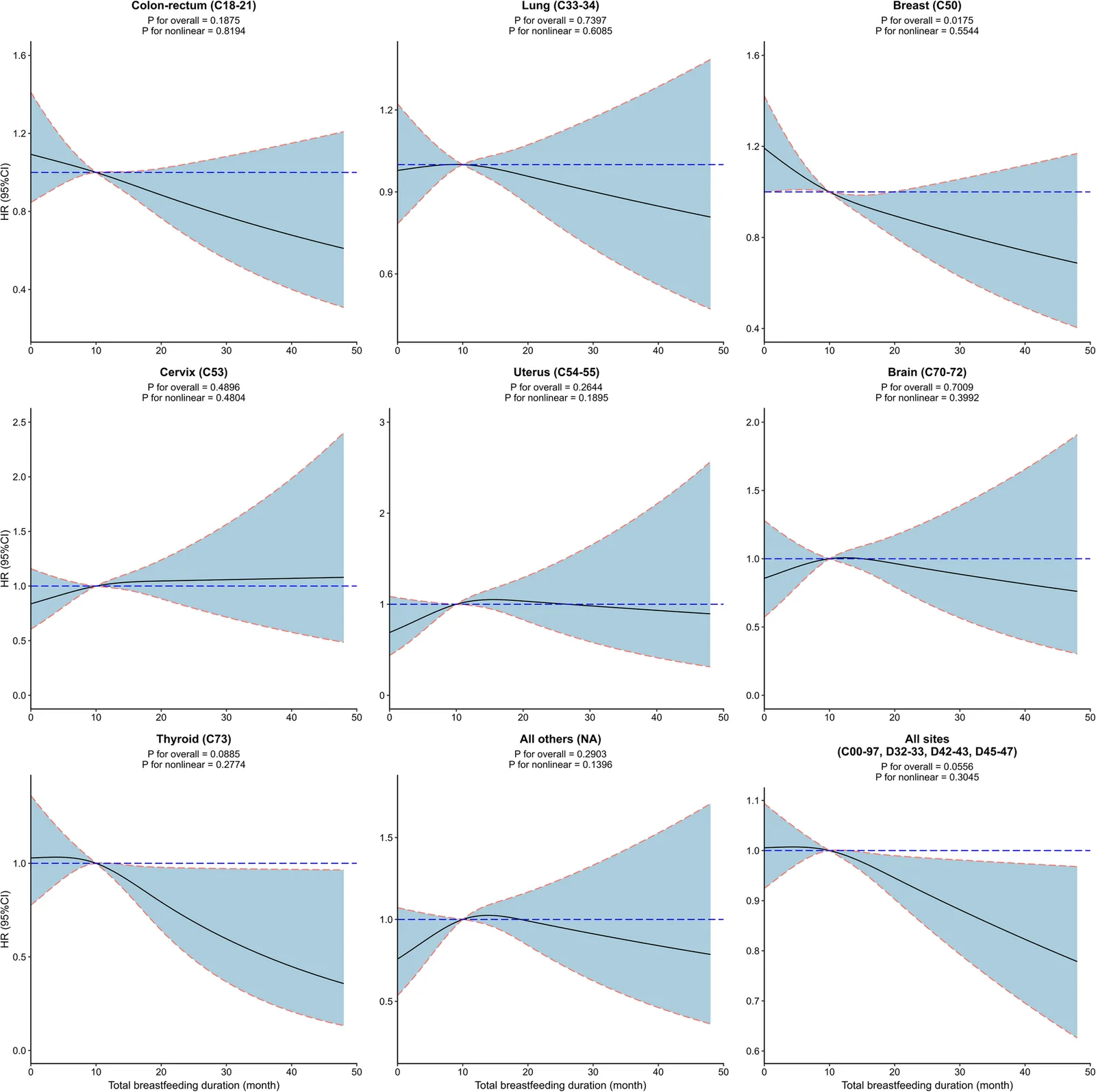
The dose-response relationship of total breastfeeding duration with the risk of overall cancer and its main site-specific cancers

Figure 2 and eFigure 2 illustrates the dose-response associations between total breastfeeding duration and the risks of overall cancer and site-specific cancers. Significant linear associations were suggested concerning gallbladder cancer (*P*-overall = 0.0302, *P*-nonlinear = 0.6558) and breast cancer (*P*-overall = 0.0175, *P*-nonlinear = 0.5544).

The results of a series of sensitivity analyses are presented in the Supplementary Material. First, breastfeeding behaviors were still significantly associated with a higher risk of gallbladder cancer and a lower risk of breast cancer, based on the HRs (99%CI) and/or FDR-adjusted *P*-values (see eTables 5–8). In contrast, the associations of breastfeeding behaviors with thyroid cancer and overall cancer incidence became marginally non-significant after stringent correction. Second, the estimated effects of breastfeeding behavior on cancer incidence remained fairly robust after additionally adjusting for surgeries on the reproductive system. That was to say, the associations between total breastfeeding duration and gallbladder cancer (HR = 1.66, 95%CI: 1.22–2.25), breast cancer (HR = 0.88, 95%CI: 0.79–0.99), and thyroid cancer (HR = 0.82, 95%CI: 0.68–0.99) remained statistically significant (see eTable 9). In the sensitivity analyses for including nulliparous female participants into analysis, the breastfeeding behavior was still associated with higher risk of gallbladder cancer and lower risk of breast cancer and thyroid cancer (see eTable 10). Additionally, history of breastfeeding was associated with nasopharynx cancer (HR = 0.45, 95%CI: 0.23–0.87), and total breastfeeding duration was associated with overall cancer (HR = 0.94, 95%CI: 0.90–0.98).

## Discussion

### Main findings

Evidence on the effect of breastfeeding on certain maternal cancers remains insufficient and controversial. To the best of our knowledge, this study is the first in China to systematically and comprehensively investigate the relationship between breastfeeding behaviors and all site-specific maternal cancers based on a large-scale population-based cohort, providing valuable evidence for the promotion of women’s health in China and Asia. In this prospective study that included 157,454 participants and 5,542 cancer cases, we discovered that the history of breastfeeding was statistically significantly associated with lower risk of breast cancer. In the multivariate-adjusted model, longer durations of total breastfeeding were associated with increased risk of gallbladder cancer and decreased risks of breast cancer, thyroid cancer, and overall cancer. The subgroup analyses according to menopausal status showed significant separations for colon-rectum cancer, uterus cancer, oesophagus cancer, and larynx cancer. In addition, significant linear associations were suggested concerning gallbladder cancer and breast cancer based on restricted cubic spline model. Our findings, derived from a relatively large population study, are highly pertinent to shaping public health policy and practices in China.

### Interpretation and comparison with previous studies

The observed association between breastfeeding and decreased risk of breast cancer was in line with previous studies. [5, 21–25] In fact, the protective effects of breastfeeding against breast cancer have been widely acknowledged by the academic researchers, whereas the precise mechanism remains uncertain. A recent review indicated that the mechanism may include the reduction of DNA methylation abnormalities, remodeling of breast tissue, lowering of estrogen levels, and inhibition of cancer cells within the microenvironment through breastfeeding. [5] In addition, the beneficial effect of breastfeeding varied for different types of breast cancer. [26] For example, findings from the Nurses’ Health Studies revealed that breastfeeding was solely associated with a reduced risk of estrogen receptor negative disease, but not estrogen receptor positive disease. [27] The varying associations between breastfeeding and breast cancer subtypes warrant further investigation, which will be addressed in our subsequent studies.

This study found that the risk of gallbladder cancer increased with increasing months of total breastfeeding (*P*-trend = 0.039), with a 51% increase in risk for each additional 12 months of breastfeeding (HR = 1.51, 95%CI: 1.12–2.02). Our results were inconsistent with the previous literature, which did not find an association between breastfeeding and gallbladder cancer. [28–31] For example, a case-control study in India discovered a positive but not statistically significant association of breastfeeding years with gallbladder cancer (OR = 1.01, 95%CI: 0.98–1.04). [30] According to a pooled analysis of the Asia Cohort Consortium across 4 countries (China, Japan, Korea, and Singapore), history of breastfeeding did not show a clear association with gallbladder cancer risk (pooled HR = 0.98, 95%CI: 0.72–1.34). [31] Despite the available literature, existing studies on the association between breastfeeding and gallbladder cancer remain limited, and further large-scale prospective studies are required to elucidate this relationship. The potential mechanism may involve breastfeeding-induced alterations in sex hormones, which are implicated in gallbladder carcinogenesis, as supported by in vitro data, animal models, and functional or physiological evidence. The abnormal expression of estrogen and progesterone receptors in gallbladder cancer tissues, vitro cell lines, and mouse models suggested a critical role for steroid hormones in the development of gallbladder carcinogenesis. [32, 33].

In this study, the risk of thyroid cancer decreased by 17% with each additional 12 months of breastfeeding (HR = 0.83, 95% CI: 0.69–0.99) in the multivariate-adjusted model, which was consistent with most previous studies. [34–37] A case-control study among Chinese women found that shorter lifetime breastfeeding (< 6 months) significantly increased the risk of papillary thyroid cancer. [34] A meta-analysis comprising nine reports revealed a significant inverse association between ever breastfeeding and thyroid cancer risk, as well as a significant linear relationship between breastfeeding duration and thyroid cancer risk. [35] Although most related studies have reported significant findings, a longitudinal study did not observe a statistically significant association regarding the months of breastfeeding and the risk of thyroid cancer. [38] The possible protective effect of breastfeeding against thyroid cancer deserves further exploration, with several potential biological mechanisms already having been suggested. Breastfeeding may inhibit ovulation, thereby reducing endogenous estrogen exposure, which in turn could decrease the proliferation, migration, invasion, and recurrence of malignant thyroid cells. [39].

This study also found a reduced risk of overall cancer with each additional 12 months of breastfeeding (HR = 0.95, 95%CI: 0.90–0.99). This suggested that breastfeeding in general may be feasible for reducing the risk of cancer among parous women, despite its potential adverse effect on gallbladder cancer. Regarding site-specific cancers, we did not discover statistically significant associations between breastfeeding and cancers other than breast, gallbladder, and thyroid cancer. In fact, previous studies have reported rather mixed results related to other cancers. For example, numerous studies have evaluated the association between breastfeeding and ovarian cancer risk, with some indicating a significant reduction in risk while others reported no association. [7, 40, 41] Similarly, the inconsistent associations were observed between breastfeeding and endometrial cancer, [42, 43] esophageal cancer, [44, 45] gastric cancer, [46, 47] colorectal cancer, [10, 48] pancreatic cancer, [49, 50] and lung cancer. [11, 17, 51] This may be attributable to variations in the study population (e.g., race, age, region), study methodology (e.g., statistical analysis methods, covariates adjusted), and cancer characteristics (e.g., anatomic subsite, histologic type, aggressiveness).

In this study, the modifying effects of menopausal status were observed in the relationships between breastfeeding and colon-rectal cancer, uterine cancer, esophageal cancer, and laryngeal cancer. The potential protective effects of breastfeeding behavior against colon-rectal cancer and uterine cancer were more pronounced in premenopausal women compared to postmenopausal women, which was in line with reports from previous studies to some extent. For instance, a systematic review and meta-analysis found breastfeeding women had a lower risk of premenopausal breast cancer but not of postmenopausal breast cancer. [52] Siskind et al. found an association between breastfeeding and a reduced risk of ovarian cancer, but only before menopause. [53] A pooled analysis from the international lung cancer consortium demonstrated a statistically significant association between breastfeeding history and lung cancer risk, exclusively in premenopausal women, where the inverse relationship was also notably stronger (*P* for interaction = 0.04). [54] The potential explanation is that breastfeeding influences cancer risk primarily through hormonal changes, with premenopausal women being more sensitive to these fluctuations and thus more likely to benefit from breastfeeding. [55] Notably, more pronounced inverse associations were observed among postmenopausal women for oesophagus cancer and larynx cancer. However, these findings should be interpreted with great caution due to the extremely limited number of cancer cases, necessitating a longer follow-up period to validate their robustness.

Restricted cubic spline model in our study demonstrated significant linear associations between total breastfeeding duration and gallbladder cancer and breast cancer, which were comparable to previous studies. The existing evidence of dose-response relationship of breastfeeding and breast cancer was inconsistent. [56–58] Shantakumar et al. found that there was a strong dose-response relationship for longer durations of breastfeeding and reduced risks of breast cancer (*P* for trend = 0.02) among older postmenopausal women. [57] A case-control study conducted among Sri Lankan women indicated that the reduction of breast cancer risk continued to increase up to the lifetime breastfeeding duration of 36–47 months and decreased thereafter. [58] With respect to gallbladder cancer, there was limited evidence for the dose-response relationship regarding duration of breastfeeding. In addition, we observed a linear association between breastfeeding duration and thyroid cancer (*P* for nonlinear > 0.05) although the overall association was borderline non-significant (*P* for overall > 0.05). Our findings were consistent with a previous dose-response meta-analysis that revealed a significant linear relationship between breastfeeding duration and risk of thyroid cancer, especially in cohort studies. [35].

### Strengths and limitations of this study

This study has several strengths. Firstly, to our knowledge, this study is the first in China to systematically examine the association of breastfeeding with all site-specific cancers, which could facilitate the exploration of less prevalent cancers and mitigate publication bias. Secondly, based on a large-scale prospective cohort derived from CanSPUC in Hunan Province, we were able to investigate the relationship between breastfeeding and maternal cancers within a representative provincial population, thereby contributing to the scientific evidence available from China and East Asia. Finally, we conducted a comprehensive statistical analysis, including a restricted cubic spline analysis to evaluate the dose-response relationship between breastfeeding and cancer, as well as subgroup and sensitivity analyses to ensure the robustness and reliability of the primary findings.

This study also has limitations. Firstly, while this cohort provides robust socioeconomic and health data, it lacks detailed reproductive histories (including parity, age at first/last birth) and dietary measurements. Although we have adjusted for some available confounders like smoking and BMI, the absence of these variables (especially parity) may lead to residual confounding. Parity information was not systematically collected in our cohort during the initial study phase, precluding adjustment for this potential confounder in breastfeeding-cancer associations. To address this gap, we have initiated systematic collection of parity data in our ongoing follow-up assessments. However, given the cohort’s substantial size and the need for rigorous quality control, this data collection and validation process will require several years to complete. Future studies incorporating reproductive calendars and nutritional assessments would help clarify these relationships. Secondly, the relatively short follow-up period resulted in a small number of cases for certain sub-specific cancers, which affected the statistical power and led to unstable results. Continued follow-up of the cohort will yield updated data, which we will analyze and present in future reports. In addition, the attenuation of associations for thyroid cancer and overall cancer incidence highlights the importance of accounting for multiple testing in large-scale analyses. Future studies with larger samples or longer follow-ups could clarify whether these trends reflect true associations or noise. Finally, despite the adequate sample size, the study’s confinement to a single province may limit the generalizability of the findings, which should therefore be interpreted with caution. Additional studies on diverse provinces and regions are necessary in the future.

### Implications and recommendations

This study mainly assessed the association between breastfeeding behaviors and the subsequent risk of 24 types of maternal cancer among parous Chinese women. The observed complex findings provide a more comprehensive insights into the prevention and control of cancer. In this study, breastfeeding appears to be associated with a reduced risk of developing cancer in general, as well as the two most common female cancers (breast and thyroid), suggesting that breastfeeding has overall beneficial effects on women. The potential adverse effects on gallbladder cancer should be further elucidated and compared with other large prospective studies in the future.

Based on the overall benefits of breastfeeding, there are several inspirations and recommendations for the prevention and control of cancer. Firstly, academic researchers and scientists could consider incorporating breastfeeding into broader cancer prevention recommendations, extending beyond just breast cancer. Guidelines for preventing specific cancers, such as thyroid cancer, may also benefit from including breastfeeding as a key recommendation. Secondly, playing a critical role in enhancing women’s health, governments are able to improve policies and programs that advocate for breastfeeding support. Finally, individuals can prevent their cancer risks by actively engaging in self-management practices related to breastfeeding, such as seeking education on proper techniques, maintaining a balanced diet during lactation, and accessing social support networks to navigate challenges.

Our study participants were drawn from the Urban Cancer Screening Program in China, a population profoundly shaped by the nation’s unique One-Child Policy (1979–2015), which may result in a proportion of urban women having only one child. [59, 60] This historical context creates a distinctive research setting where near-universal single-child status minimizes parity-related confounding—a persistent challenge in Western cohorts with wider parity variation. Although we lack individual parity data for stratified analysis, the policy-induced homogeneity in reproductive behavior ensures that breastfeeding effects are less likely to be distorted by multiparity interactions. These findings hold significant relevance for contemporary China, where delayed childbearing and low fertility persist, [61] and they provide unique insights into cancer prevention in populations with constrained parity variability.

## Conclusions

In the large-scale population-based cohort study, we found that breastfeeding behaviors were associated with lower risk of breast cancer and thyroid cancer, and increased risk of gallbladder cancer. Besides, significant linear associations were suggested concerning breast cancer and gallbladder cancer. We also provided novel evidence of a more pronounced protective effect of breastfeeding behavior against two common cancers, including colorectal cancer and uterine cancer, in premenopausal women. These findings will benefit policy-making and intervention design in cancer prevention and suggest that the underlying biological mechanisms of breastfeeding related to cancer need to be revealed urgently.

## Supplementary Information

Supplementary Material 1

## Data Availability

The data will be available from the corresponding authors on request.
