# Supplementary material for: Breastfeeding and subsequent risk of 24 types of maternal cancer: a cohort study of Chinese women
Source: Nutr J. 2026 Jun 11;25:94. doi: 10.1186/s12937-026-01346-2 (PMC13487942; doi:10.1186/s12937-026-01346-2)
Supplement: Supplementary file 1 [file 12937_2026_1346_MOESM1_ESM.docx]

**eFigure 1. The flow chart of the included participants in this study.**

**eFigure 2. The dose-response relationship of total breastfeeding duration with the risk of rare site-specific cancers.**

**eTable 1. The definition and number of overall cancer and its specific-site cancers.**

**eTable 2. Risk of developing** **rare site-specific cancers in relationship to history of breastfeeding.**

**eTable 3. Risk of developing rare site-specific cancers in relationship to baseline total breastfeeding duration.**

**eTable 4. Risk of developing rare site-specific cancers in relationship to breastfeeding behavior stratified by menopausal status.**

**eTable 5. Sensitivity analysis for risk of developing overall cancer and its main site-specific cancers in relationship to history of breastfeeding.
eTable 6. Sensitivity analysis for risk of developing rare site-specific cancers in relationship to history of breastfeeding.**
**eTable 7. Sensitivity analysis for risk of developing overall cancer and its main site-specific cancers in relationship to baseline total breastfeeding duration.**
**eTable 8. Sensitivity analysis for risk of developing rare site-specific cancers in relationship to baseline total breastfeeding duration.**

**eTable 9. Sensitivity analysis for further adjusting for surgeries on the reproductive system among parous women (2012-2019).**

**eTable 10. Sensitivity analysis for including nulliparous female participants.**

**eFigure 1. The flow chart of the included participants in this study.**

**eFigure 2. The dose-response relationship of total breastfeeding duration with the risk of rare site-specific cancers.**

**eTable 1. The definition and number of overall cancer and its specific-site cancers.**

|  | **ICD-10** | **n** |
| --- | --- | --- |
| Non-cancer | - | 151912 |
| Oral cavity and pharynx | C00-10, C12-14 | 69 |
| Nasopharynx | C11 | 49 |
| Oesophagus | C15 | 27 |
| Stomach | C16 | 165 |
| Colon-rectum | C18-21 | 574 |
| Liver | C22 | 190 |
| Gallbladder | C23-24 | 84 |
| Pancreas | C25 | 68 |
| Larynx | C32 | 14 |
| Lung | C33-34 | 804 |
| Other thoracic organs | C37-38 | 24 |
| Bone | C40-41 | 23 |
| Melanoma of skin | C43 | 26 |
| Breast | C50 | 1128 |
| Cervix | C53 | 407 |
| Uterus | C54-55 | 240 |
| Ovary | C56 | 135 |
| Prostate | C61 | - |
| Testis | C62 | - |
| Kidney | C64-66, C68 | 87 |
| Bladder | C67 | 50 |
| Brain | C70-72 | 271 |
| Thyroid | C73 | 472 |
| Lymphoma | C81-85, C88, C90, C96 | 157 |
| Leukaemia | C91-95 | 86 |
| All others | NA | 392 |
| All sites | C00-97, D32-33, D42-43, D45-47 | 5542 |

**eTable 2. Risk of developing rare site-specific cancers in relationship to history of breastfeeding.**

| **History of breastfeeding** | **Cases (n=5542)** | **Unadjusted model** |  | **Age-adjusted model** |  | **Multivariate-adjusted model** |
| --- | --- | --- | --- | --- | --- | --- |
|  |  | **HR (95%CI)** |  | **HR (95%CI)** |  | **HR (95%CI)** |
| Oral cavity and pharynx (C00-10, C12-14) |  |  |  |  |  |  |
| No | 4 | Ref. |  | Ref. |  | Ref. |
| Yes | 65 | 1.48 (0.54-4.07) |  | 1.45 (0.53-3.97) |  | 1.39 (0.50-3.86) |
| Nasopharynx (C11) |  |  |  |  |  |  |
| No | 8 | Ref. |  | Ref. |  | Ref. |
| No | 41 | 0.49 (0.23-1.04) |  | 0.49 (0.23-1.04) |  | 0.53 (0.24-1.16) |
| Oesophagus (C15) |  |  |  |  |  |  |
| No | 1 | Ref. |  | Ref. |  | Ref. |
| Yes | 26 | 2.47 (0.33-18.21) |  | 2.29 (0.31-16.87) |  | 2.57 (0.34-19.22) |
| Stomach (C16) |  |  |  |  |  |  |
| No | 10 | Ref. |  | Ref. |  | Ref. |
| Yes | 155 | 1.45 (0.77-2.76) |  | 1.40 (0.74-2.65) |  | 1.51 (0.79-2.89) |
| Liver (C22) |  |  |  |  |  |  |
| No | 18 | Ref. |  | Ref. |  | Ref. |
| Yes | 172 | 0.89 (0.55-1.45) |  | 0.86 (0.53-1.39) |  | 0.89 (0.54-1.46) |
| Gallbladder (C23-24) |  |  |  |  |  |  |
| No | 5 | Ref. |  | Ref. |  | Ref. |
| Yes | 79 | 1.45 (0.59-3.57) |  | 1.35 (0.55-3.34) |  | 1.51 (0.60-3.76) |
| Pancreas (C25) |  |  |  |  |  |  |
| No | 8 | Ref. |  | Ref. |  | Ref. |
| Yes | 60 | 0.68 (0.33-1.43) |  | 0.63 (0.30-1.32) |  | 0.58 (0.28-1.23) |
| Larynx (C32) |  |  |  |  |  |  |
| No | 1 | Ref. |  | Ref. |  | Ref. |
| Yes | 13 | 1.21 (0.16-9.30) |  | 1.15 (0.15-8.82) |  | 1.38 (0.17-11.07) |
| Other thoracic organs (C37-38) |  |  |  |  |  |  |
| No | 1 | Ref. |  | Ref. |  | Ref. |
| Yes | 23 | 2.10 (0.28-15.57) |  | 1.98 (0.27-14.70) |  | 2.22 (0.30-16.73) |
| Bone (C40-41) |  |  |  |  |  |  |
| No | 2 | Ref. |  | Ref. |  | Ref. |
| Yes | 21 | 0.94 (0.22-4.02) |  | 0.89 (0.21-3.81) |  | 0.83 (0.19-3.60) |
| Melanoma of skin (C43) |  |  |  |  |  |  |
| No | 4 | Ref. |  | Ref. |  | Ref. |
| Yes | 22 | 0.52 (0.18-1.51) |  | 0.50 (0.17-1.44) |  | 0.53 (0.18-1.59) |
| Ovary (C56) |  |  |  |  |  |  |
| No | 10 | Ref. |  | Ref. |  | Ref. |
| Yes | 125 | 1.14 (0.60-2.18) |  | 1.14 (0.60-2.17) |  | 1.19 (0.62-2.29) |
| Kidney (C64-66, C68) |  |  |  |  |  |  |
| No | 9 | Ref. |  | Ref. |  | Ref. |
| Yes | 78 | 0.79 (0.40-1.58) |  | 0.75 (0.38-1.50) |  | 0.80 (0.40-1.62) |
| Bladder (C67) |  |  |  |  |  |  |
| No | 2 | Ref. |  | Ref. |  | Ref. |
| Yes | 48 | 2.18 (0.53-8.99) |  | 2.04 (0.50-8.41) |  | 2.17 (0.52-9.02) |
| Lymphoma (C81-85, C88, C90, C96) |  |  |  |  |  |  |
| No | 14 | Ref. |  | Ref. |  | Ref. |
| Yes | 143 | 0.94 (0.54-1.63) |  | 0.90 (0.52-1.55) |  | 0.91 (0.52-1.58) |
| Leukaemia (C91-95) |  |  |  |  |  |  |
| No | 4 | Ref. |  | Ref. |  | Ref. |
| Yes | 82 | 1.87 (0.69-5.12) |  | 1.81 (0.66-4.93) |  | 1.87 (0.68-5.13) |

Unadjusted model: no covariates were controlled for.

Age-adjusted model: only age at enrollment (continuous) was controlled for.

Multivariate-adjusted model: age-adjusted model + educational level (low, medium, high), marital status (married, others), smoking status (never, current, ever), alcohol drinking status (never, ever), regular exercise (no, yes), BMI (underweight, normal, overweight, obese), family history of cancer (no, yes), hypertension (no, yes), diabetes (no, yes), age at menarche (<13, 13, 14, >14 years), and menopause status (no, yes).

Abbreviations: HR, hazard ratio; CI, confidence interval.

**eTable 3. Risk of developing rare site-specific cancers in relationship to baseline total breastfeeding duration.**

| **Site-specific cancers** | **Total breastfeeding duration** | **Cases**  **(n=5542)** | **Unadjusted model** | **Age-adjusted model** | **Multivariate-adjusted model** |
| --- | --- | --- | --- | --- | --- |
|  |  |  | **HR (95%CI)** | **HR (95%CI)** | **HR (95%CI)** |
| Oral cavity and pharynx (C00-10, C12-14) | Continuous (per 12 months) | 69 | 0.80 (0.51-1.26) | 0.75 (0.48-1.17) | 0.70 (0.44-1.12) |
|  | Multi-categorical (month) |  |  |  |  |
|  | 0 | 4 | Ref. | Ref. | Ref. |
|  | 1-6 | 11 | 1.95 (0.62-6.14) | 1.97 (0.63-6.20) | 1.99 (0.63-6.28) |
|  | 7-12 | 42 | 1.46 (0.52-4.07) | 1.45 (0.52-4.04) | 1.38 (0.49-3.89) |
|  | >12 | 12 | 1.26 (0.41-3.92) | 1.15 (0.37-3.58) | 1.08 (0.34-3.40) |
|  | *P* for trend |  | 0.874 | 0.706 | 0.590 |
| Nasopharynx (C11) | Continuous (per 12 months) | 49 | 1.03 (0.63-1.67) | 1.02 (0.62-1.67) | 0.98 (0.61-1.59) |
|  | Multi-categorical (month) |  |  |  |  |
|  | 0 | 8 | Ref. | Ref. | Ref. |
|  | 1-6 | 6 | 0.56 (0.19-1.61) | 0.56 (0.19-1.61) | 0.60 (0.21-1.75) |
|  | 7-12 | 24 | 0.43 (0.19-0.97) | 0.43 (0.19-0.97) | 0.49 (0.22-1.12) |
|  | >12 | 11 | 0.61 (0.24-1.51) | 0.61 (0.24-1.51) | 0.61 (0.24-1.56) |
|  | *P* for trend |  | 0.259 | 0.253 | 0.283 |
| Oesophagus (C15) | Continuous (per 12 months) | 27 | 1.07 (0.57-2.03) | 0.90 (0.48-1.71) | 0.98 (0.51-1.88) |
|  | Multi-categorical (month) |  |  |  |  |
|  | 0 | 1 | Ref. | Ref. | Ref. |
|  | 1-6 | 3 | 2.21 (0.23-21.28) | 2.27 (0.24-21.84) | 2.38 (0.25-23.04) |
|  | 7-12 | 19 | 2.77 (0.37-20.69) | 2.66 (0.36-19.89) | 2.96 (0.39-22.49) |
|  | >12 | 4 | 1.73 (0.19-15.52) | 1.37 (0.15-12.29) | 1.62 (0.18-14.81) |
|  | *P* for trend |  | 0.729 | 0.982 | 0.834 |
| Stomach (C16) | Continuous (per 12 months) | 165 | 1.13 (0.87-1.45) | 1.01 (0.78-1.30) | 1.03 (0.80-1.34) |
|  | Multi-categorical (month) |  |  |  |  |
|  | 0 | 10 | Ref. | Ref. | Ref. |
|  | 1-6 | 18 | 1.32 (0.61-2.87) | 1.35 (0.62-2.93) | 1.44 (0.66-3.14) |
|  | 7-12 | 103 | 1.48 (0.77-2.83) | 1.46 (0.76-2.79) | 1.57 (0.81-3.04) |
|  | >12 | 34 | 1.45 (0.72-2.93) | 1.26 (0.62-2.55) | 1.37 (0.67-2.82) |
|  | *P* for trend |  | 0.321 | 0.654 | 0.497 |
| Liver (C22) | Continuous (per 12 months) | 190 | 0.93 (0.72-1.20) | 0.82 (0.64-1.06) | 0.79 (0.61-1.03) |
|  | Multi-categorical (month) |  |  |  |  |
|  | 0 | 18 | Ref. | Ref. | Ref. |
|  | 1-6 | 30 | 1.22 (0.68-2.18) | 1.25 (0.70-2.24) | 1.32 (0.73-2.38) |
|  | 7-12 | 100 | 0.79 (0.48-1.30) | 0.78 (0.47-1.29) | 0.81 (0.49-1.35) |
|  | >12 | 42 | 1.02 (0.59-1.77) | 0.87 (0.50-1.51) | 0.86 (0.49-1.51) |
|  | *P* for trend |  | 0.562 | 0.209 | 0.192 |
| Gallbladder (C23-24) | Continuous (per 12 months) | 84 | 1.82 (1.36-2.42) | 1.54 (1.15-2.06) | 1.51 (1.12-2.02) |
|  | Multi-categorical (month) |  |  |  |  |
|  | 0 | 5 | Ref. | Ref. | Ref. |
|  | 1-6 | 8 | 1.14 (0.37-3.49) | 1.17 (0.38-3.59) | 1.13 (0.36-3.59) |
|  | 7-12 | 42 | 1.17 (0.46-2.97) | 1.14 (0.45-2.88) | 1.33 (0.52-3.41) |
|  | >12 | 29 | 2.46 (0.95-6.37) | 1.98 (0.77-5.14) | 2.19 (0.83-5.76) |
|  | *P* for trend |  | 0.013 | 0.062 | 0.039 |
| Pancreas (C25) | Continuous (per 12 months) | 68 | 1.25 (0.85-1.84) | 1.02 (0.70-1.49) | 0.94 (0.63-1.41) |
|  | Multi-categorical (month) |  |  |  |  |
|  | 0 | 8 | Ref. | Ref. | Ref. |
|  | 1-6 | 8 | 0.71 (0.27-1.88) | 0.74 (0.28-1.97) | 0.74 (0.28-1.99) |
|  | 7-12 | 37 | 0.64 (0.30-1.38) | 0.62 (0.29-1.33) | 0.58 (0.27-1.25) |
|  | >12 | 15 | 0.80 (0.34-1.88) | 0.61 (0.26-1.45) | 0.52 (0.22-1.25) |
|  | *P* for trend |  | 0.654 | 0.264 | 0.129 |
| Larynx (C32) | Continuous (per 12 months) | 14 | 0.87 (0.33-2.26) | 0.77 (0.30-1.98) | 0.75 (0.29-1.92) |
|  | Multi-categorical (month) |  |  |  |  |
|  | 0 | 1 | Ref. | Ref. | Ref. |
|  | 1-6 | 3 | 2.21 (0.23-21.27) | 2.26 (0.23-21.73) | 2.87 (0.29-28.54) |
|  | 7-12 | 6 | 0.86 (0.10-7.18) | 0.84 (0.10-7.01) | 1.02 (0.12-8.85) |
|  | >12 | 4 | 1.68 (0.19-15.03) | 1.41 (0.16-12.73) | 1.54 (0.16-14.61) |
|  | *P* for trend |  | 0.959 | 0.885 | 0.898 |
| Other thoracic organs (C37-38) | Continuous (per 12 months) | 24 | 1.14 (0.58-2.24) | 0.98 (0.50-1.91) | 1.15 (0.58-2.30) |
|  | Multi-categorical (month) |  |  |  |  |
|  | 0 | 1 | Ref. | Ref. | Ref. |
|  | 1-6 | 4 | 2.84 (0.32-25.41) | 2.92 (0.33-26.19) | 2.70 (0.30-24.37) |
|  | 7-12 | 13 | 1.81 (0.24-13.80) | 1.76 (0.23-13.50) | 1.96 (0.25-15.23) |
|  | >12 | 6 | 2.58 (0.31-21.42) | 2.10 (0.25-17.50) | 2.64 (0.31-22.55) |
|  | *P* for trend |  | 0.580 | 0.805 | 0.530 |
| Bone (C40-41) | Continuous (per 12 months) | 23 | 1.66 (0.93-2.98) | 1.45 (0.81-2.60) | 1.32 (0.72-2.42) |
|  | Multi-categorical (month) |  |  |  |  |
|  | 0 | 2 | Ref. | Ref. | Ref. |
|  | 1-6 | 3 | 1.04 (0.17-6.21) | 1.05 (0.18-6.30) | 1.01 (0.17-6.07) |
|  | 7-12 | 10 | 0.68 (0.15-3.11) | 0.67 (0.15-3.04) | 0.63 (0.14-2.95) |
|  | >12 | 8 | 1.70 (0.36-8.00) | 1.43 (0.30-6.74) | 1.24 (0.25-6.04) |
|  | *P* for trend |  | 0.422 | 0.588 | 0.731 |
| Melanoma of skin (C43) | Continuous (per 12 months) | 26 | 0.78 (0.38-1.63) | 0.69 (0.34-1.43) | 0.72 (0.34-1.52) |
|  | Multi-categorical (month) |  |  |  |  |
|  | 0 | 4 | Ref. | Ref. | Ref. |
|  | 1-6 | 4 | 0.74 (0.18-2.96) | 0.76 (0.19-3.05) | 0.78 (0.19-3.16) |
|  | 7-12 | 12 | 0.43 (0.14-1.34) | 0.43 (0.14-1.32) | 0.47 (0.15-1.52) |
|  | >12 | 6 | 0.65 (0.18-2.31) | 0.55 (0.15-1.96) | 0.52 (0.13-2.03) |
|  | *P* for trend |  | 0.377 | 0.253 | 0.248 |
| Ovary (C56) | Continuous (per 12 months) | 135 | 0.81 (0.59-1.12) | 0.80 (0.58-1.11) | 0.83 (0.60-1.15) |
|  | Multi-categorical (month) |  |  |  |  |
|  | 0 | 10 | Ref. | Ref. | Ref. |
|  | 1-6 | 21 | 1.49 (0.70-3.17) | 1.49 (0.70-3.17) | 1.50 (0.70-3.19) |
|  | 7-12 | 87 | 1.21 (0.63-2.33) | 1.21 (0.63-2.33) | 1.26 (0.65-2.44) |
|  | >12 | 17 | 0.72 (0.33-1.58) | 0.71 (0.32-1.56) | 0.75 (0.34-1.66) |
|  | *P* for trend |  | 0.199 | 0.185 | 0.273 |
| Kidney (C64-66, C68) | Continuous (per 12 months) | 87 | 1.15 (0.81-1.64) | 1.01 (0.72-1.44) | 1.03 (0.72-1.47) |
|  | Multi-categorical (month) |  |  |  |  |
|  | 0 | 9 | Ref. | Ref. | Ref. |
|  | 1-6 | 4 | 0.32 (0.10-1.02) | 0.32 (0.10-1.04) | 0.34 (0.11-1.12) |
|  | 7-12 | 55 | 0.85 (0.42-1.72) | 0.83 (0.41-1.69) | 0.90 (0.44-1.84) |
|  | >12 | 19 | 0.89 (0.40-1.97) | 0.75 (0.34-1.67) | 0.81 (0.36-1.82) |
|  | *P* for trend |  | 0.538 | 0.890 | 0.797 |
| Bladder (C67) | Continuous (per 12 months) | 50 | 1.33 (0.86-2.05) | 1.13 (0.73-1.73) | 1.18 (0.76-1.83) |
|  | Multi-categorical (month) |  |  |  |  |
|  | 0 | 2 | Ref. | Ref. | Ref. |
|  | 1-6 | 6 | 2.13 (0.43-10.58) | 2.19 (0.44-10.86) | 2.17 (0.44-10.79) |
|  | 7-12 | 28 | 1.95 (0.46-8.19) | 1.89 (0.45-7.95) | 2.02 (0.48-8.57) |
|  | >12 | 14 | 2.92 (0.66-12.85) | 2.35 (0.53-10.38) | 2.59 (0.58-11.60) |
|  | *P* for trend |  | 0.149 | 0.334 | 0.248 |
| Lymphoma (C81-85, C88, C90, C96) | Continuous (per 12 months) | 157 | 1.01 (0.77-1.33) | 0.88 (0.67-1.16) | 0.90 (0.68-1.20) |
|  | Multi-categorical (month) |  |  |  |  |
|  | 0 | 14 | Ref. | Ref. | Ref. |
|  | 1-6 | 18 | 0.92 (0.46-1.86) | 0.94 (0.47-1.90) | 0.98 (0.48-1.97) |
|  | 7-12 | 93 | 0.94 (0.53-1.64) | 0.92 (0.52-1.61) | 0.91 (0.52-1.61) |
|  | >12 | 32 | 0.98 (0.52-1.84) | 0.82 (0.44-1.54) | 0.86 (0.45-1.62) |
|  | *P* for trend |  | 0.988 | 0.508 | 0.575 |
| Leukaemia (C91-95) | Continuous (per 12 months) | 86 | 1.23 (0.87-1.73) | 1.11 (0.78-1.56) | 1.13 (0.80-1.61) |
|  | Multi-categorical (month) |  |  |  |  |
|  | 0 | 4 | Ref. | Ref. | Ref. |
|  | 1-6 | 9 | 1.60 (0.49-5.20) | 1.63 (0.50-5.28) | 1.61 (0.50-5.26) |
|  | 7-12 | 56 | 1.96 (0.71-5.40) | 1.93 (0.70-5.32) | 2.00 (0.72-5.57) |
|  | >12 | 17 | 1.79 (0.60-5.32) | 1.56 (0.52-4.65) | 1.65 (0.55-4.97) |
|  | *P* for trend |  | 0.320 | 0.536 | 0.440 |

Unadjusted model: no covariates were controlled for.

Age-adjusted model: only age at enrollment (continuous) was controlled for.

Multivariate-adjusted model: age-adjusted model + educational level (low, medium, high), marital status (married, others), smoking status (never, current, ever), alcohol drinking status (never, ever), regular exercise (no, yes), BMI (underweight, normal, overweight, obese), family history of cancer (no, yes), hypertension (no, yes), diabetes (no, yes), age at menarche (<13, 13, 14, >14 years), and menopause status (no, yes).

Abbreviations: HR, hazard ratio; CI, confidence interval.

**eTable 4. Risk of developing rare site-specific cancers in relationship to breastfeeding behavior stratified by menopausal status.**

| **Site-specific cancers** | **Breastfeeding behavior** | **Premenopausal** | | **Postmenopausal** | | ***P*_int_** |
| --- | --- | --- | --- | --- | --- | --- |
|  |  | **Cases** | **HR (95%CI)** | **Cases** | **HR (95%CI)** |  |
| Oral cavity and pharynx (C00-10, C12-14) |  |  |  |  |  |  |
|  | History of breastfeeding |  |  |  |  |  |
|  | No | 1 | Ref. | 3 | Ref. |  |
|  | Yes | 18 | 1.70 (0.22-12.93) | 47 | 1.26 (0.39-4.09) | 0.755 |
|  | Total breastfeeding duration  (per 12 months) | 19 | 0.96 (0.36-2.52) | 50 | 0.64 (0.38-1.09) | 0.461 |
| Nasopharynx (C11) |  |  |  |  |  |  |
|  | History of breastfeeding |  |  |  |  |  |
|  | No | 3 | Ref. | 5 | Ref. |  |
|  | Yes | 9 | 0.30 (0.08-1.16) | 32 | 0.67 (0.26-1.77) | 0.445 |
|  | Total breastfeeding duration  (per 12 months) | 12 | 0.91 (0.25-3.38) | 37 | 0.97 (0.58-1.63) | 0.865 |
| Oesophagus (C15) |  |  |  |  |  |  |
|  | History of breastfeeding |  |  |  |  |  |
|  | No | 0 | Ref. | 1 | Ref. |  |
|  | Yes | 3 | NA | 23 | 2.23 (0.29-16.84) | 0.998 |
|  | Total breastfeeding duration  (per 12 months) | 3 | 6.01 (1.46-24.70) | 24 | 0.79 (0.38-1.65) | 0.037 |
| Stomach (C16) |  |  |  |  |  |  |
|  | History of breastfeeding |  |  |  |  |  |
|  | No | 2 | Ref. | 8 | Ref. |  |
|  | Yes | 26 | 1.52 (0.36-6.51) | 129 | 1.51 (0.73-3.11) | 0.993 |
|  | Total breastfeeding duration  (per 12 months) | 28 | 1.26 (0.60-2.65) | 137 | 1.00 (0.76-1.32) | 0.676 |
| Liver (C22) |  |  |  |  |  |  |
|  | History of breastfeeding |  |  |  |  |  |
|  | No | 4 | Ref. | 14 | Ref. |  |
|  | Yes | 25 | 0.71 (0.24-2.06) | 147 | 0.95 (0.54-1.65) | 0.621 |
|  | Total breastfeeding duration  (per 12 months) | 29 | 0.60 (0.26-1.36) | 161 | 0.82 (0.63-1.08) | 0.474 |
| Gallbladder (C23-24) |  |  |  |  |  |  |
|  | History of breastfeeding |  |  |  |  |  |
|  | No | 1 | Ref. | 4 | Ref. |  |
|  | Yes | 7 | 0.99 (0.12-8.52) | 72 | 1.63 (0.59-4.50) | 0.579 |
|  | Total breastfeeding duration  (per 12 months) | 8 | 1.21 (0.36-4.07) | 76 | 1.55 (1.14-2.10) | 0.719 |
| Pancreas (C25) |  |  |  |  |  |  |
|  | History of breastfeeding |  |  |  |  |  |
|  | No | 1 | Ref. | 7 | Ref. |  |
|  | Yes | 3 | 0.33 (0.03-3.17) | 57 | 0.61 (0.27-1.34) | 0.584 |
|  | Total breastfeeding duration  (per 12 months) | 4 | 0.15 (0.01-1.76) | 64 | 1.00 (0.67-1.49) | 0.105 |
| Larynx (C32) |  |  |  |  |  |  |
|  | History of breastfeeding |  |  |  |  |  |
|  | No | 0 | Ref. | 1 | Ref. |  |
|  | Yes | 2 | NA | 11 | 1.04 (0.13-8.38) | 0.999 |
|  | Total breastfeeding duration  (per 12 months) | 2 | 12.62 (2.69-59.25) | 12 | 0.37 (0.11-1.22) | 0.002 |
| Other thoracic organs (C37-38) |  |  |  |  |  |  |
|  | History of breastfeeding |  |  |  |  |  |
|  | No | 0 | Ref. | 1 | Ref. |  |
|  | Yes | 5 | NA | 18 | 1.58 (0.21-12.05) | 0.998 |
|  | Total breastfeeding duration  (per 12 months) | 5 | 0.64 (0.08-5.32) | 19 | 1.21 (0.58-2.50) | 0.637 |
| Bone (C40-41) |  |  |  |  |  |  |
|  | History of breastfeeding |  |  |  |  |  |
|  | No | 0 | Ref. | 2 | Ref. |  |
|  | Yes | 2 | NA | 19 | 0.74 (0.17-3.25) | 0.998 |
|  | Total breastfeeding duration  (per 12 months) | 2 | 0.77 (0.03-18.13) | 21 | 1.32 (0.72-2.44) | 0.700 |
| Melanoma of skin (C43) |  |  |  |  |  |  |
|  | History of breastfeeding |  |  |  |  |  |
|  | No | 0 | Ref. | 4 | Ref. |  |
|  | Yes | 3 | NA | 19 | 0.44 (0.15-1.35) | 0.999 |
|  | Total breastfeeding duration  (per 12 months) | 3 | 2.42 (0.20-28.63) | 23 | 0.66 (0.30-1.45) | 0.367 |
| Ovary (C56) |  |  |  |  |  |  |
|  | History of breastfeeding |  |  |  |  |  |
|  | No | 3 | Ref. | 7 | Ref. |  |
|  | Yes | 39 | 1.51 (0.46-4.96) | 86 | 1.06 (0.48-2.31) | 0.765 |
|  | Total breastfeeding duration  (per 12 months) | 42 | 0.76 (0.38-1.49) | 93 | 0.85 (0.59-1.24) | 0.674 |
| Kidney (C64-66, C68) |  |  |  |  |  |  |
|  | History of breastfeeding |  |  |  |  |  |
|  | No | 3 | Ref. | 6 | Ref. |  |
|  | Yes | 11 | 0.39 (0.11-1.39) | 67 | 1.01 (0.43-2.36) | 0.259 |
|  | Total breastfeeding duration  (per 12 months) | 14 | 0.58 (0.18-1.81) | 73 | 1.09 (0.76-1.58) | 0.342 |
| Bladder (C67) |  |  |  |  |  |  |
|  | History of breastfeeding |  |  |  |  |  |
|  | No | 0 | Ref. | 2 | Ref. |  |
|  | Yes | 6 | NA | 42 | 1.82 (0.44-7.62) | 0.997 |
|  | Total breastfeeding duration  (per 12 months) | 6 | 4.03 (1.29-12.64) | 44 | 1.02 (0.64-1.65) | 0.148 |
| Lymphoma (C81-85, C88, C90, C96) |  |  |  |  |  |  |
|  | History of breastfeeding |  |  |  |  |  |
|  | No | 1 | Ref. | 13 | Ref. |  |
|  | Yes | 25 | 2.08 (0.28-15.49) | 118 | 0.82 (0.46-1.47) | 0.250 |
|  | Total breastfeeding duration  (per 12 months) | 26 | 0.54 (0.21-1.39) | 131 | 0.95 (0.71-1.27) | 0.402 |
| Leukaemia (C91-95) |  |  |  |  |  |  |
|  | History of breastfeeding |  |  |  |  |  |
|  | No | 3 | Ref. | 1 | Ref. |  |
|  | Yes | 17 | 0.54 (0.15-1.90) | 65 | 5.53 (0.76-40.00) | 0.068 |
|  | Total breastfeeding duration  (per 12 months) | 20 | 0.93 (0.34-2.54) | 66 | 1.14 (0.79-1.66) | 0.610 |

The Cox regression model adjusted for age at enrollment (continuous), educational level (low, medium, high), marital status (married, others), smoking status (never, current, ever), alcohol drinking status (never, ever), regular exercise (no, yes), BMI (underweight, normal, overweight, obese), family history of cancer (no, yes), hypertension (no, yes), diabetes (no, yes), and age at menarche (<13, 13, 14, >14 years), and menopause status (no, yes).

Abbreviations: HR, hazard ratio; CI, confidence interval; NA, not applicable; *P*_int_, *P* for interaction effect.

**eTable 5. Sensitivity analysis for risk of developing overall cancer and its main site-specific cancers in relationship to history of breastfeeding.**

| **Main site-specific cancers** | **Multivariate-adjusted model** | | | |
| --- | --- | --- | --- | --- |
|  | **HR (95%CI)** | **HR (99%CI)** | ***P*-value** | ***P*-value _FDR_** |
| Colon-rectum (C18-21) |  |  |  |  |
| No | Ref. | Ref. |  |  |
| Yes | 1.04 (0.77-1.40) | 1.04 (0.70-1.54) | 0.811 | 0.818 |
| Lung (C33-34) |  |  |  |  |
| No | Ref. | Ref. |  |  |
| Yes | 1.07 (0.83-1.39) | 1.07 (0.77-1.51) | 0.584 | 0.791 |
| Breast (C50) |  |  |  |  |
| No | Ref. | Ref. |  |  |
| Yes | 0.78 (0.65-0.94) | 0.78 (0.61-0.99) | 0.009 | 0.081 |
| Cervix (C53) |  |  |  |  |
| No | Ref. | Ref. |  |  |
| Yes | 1.17 (0.81-1.69) | 1.17 (0.72-1.90) | 0.393 | 0.791 |
| Uterus (C54-55) |  |  |  |  |
| No | Ref. | Ref. |  |  |
| Yes | 1.14 (0.70-1.85) | 1.14 (0.60-2.16) | 0.602 | 0.791 |
| Brain (C70-72) |  |  |  |  |
| No | Ref. | Ref. |  |  |
| Yes | 1.14 (0.72-1.80) | 1.14 (0.62-2.09) | 0.585 | 0.791 |
| Thyroid (C73) |  |  |  |  |
| No | Ref. | Ref. |  |  |
| Yes | 0.92 (0.68-1.26) | 0.92 (0.62-1.39) | 0.615 | 0.791 |
| All others (NA) |  |  |  |  |
| No | Ref. | Ref. |  |  |
| Yes | 1.19 (0.81-1.76) | 1.19 (0.71-1.99) | 0.378 | 0.791 |
| All sites (C00-97, D32-33, D42-43, D45-47) |  |  |  |  |
| No | Ref. | Ref. |  |  |
| Yes | 0.99 (0.90-1.09) | 0.99 (0.87-1.12) | 0.818 | 0.818 |

Multivariate-adjusted model: age at enrollment, educational level (low, medium, high), marital status (married, others), smoking status (never, current, ever), alcohol drinking status (never, ever), regular exercise (no, yes), BMI (underweight, normal, overweight, obese), family history of cancer (no, yes), hypertension (no, yes), diabetes (no, yes), age at menarche (<13, 13, 14, >14 years), and menopause status (no, yes).

**Two methods were implemented to mitigate false positives resulting from multiple comparisons:** (i) reporting 99% confidence intervals (in addition to conventional 95%CIs) to apply more stringent significance thresholds; (ii) adjusting *P*-values using the Benjamini-Hochberg false discovery rate (FDR) correction.

Abbreviations: HR, hazard ratio; CI, confidence interval; FDR, false discovery rate.

**eTable 6. Sensitivity analysis for risk of developing rare site-specific cancers in relationship to history of breastfeeding.**

| **Rare site-specific cancers** | **Multivariate-adjusted model** | | | |
| --- | --- | --- | --- | --- |
|  | **HR (95%CI)** | **HR (99%CI)** | ***P*-value** | ***P*-value _FDR_** |
| Oral cavity and pharynx (C00-10, C12-14) |  |  |  |  |
| No | Ref. | Ref. |  |  |
| Yes | 1.39 (0.50-3.86) | 1.39 (0.37-5.32) | 0.522 | 0.779 |
| Nasopharynx (C11) |  |  |  |  |
| No | Ref. | Ref. |  |  |
| No | 0.53 (0.24-1.16) | 0.53 (0.19-1.49) | 0.114 | 0.761 |
| Oesophagus (C15) |  |  |  |  |
| No | Ref. | Ref. |  |  |
| Yes | 2.57 (0.34-19.22) | 2.57 (0.18-36.19) | 0.359 | 0.761 |
| Stomach (C16) |  |  |  |  |
| No | Ref. | Ref. |  |  |
| Yes | 1.51 (0.79-2.89) | 1.51 (0.64-3.54) | 0.213 | 0.761 |
| Liver (C22) |  |  |  |  |
| No | Ref. | Ref. |  |  |
| Yes | 0.89 (0.54-1.46) | 0.89 (0.47-1.71) | 0.648 | 0.798 |
| Gallbladder (C23-24) |  |  |  |  |
| No | Ref. | Ref. |  |  |
| Yes | 1.51 (0.60-3.76) | 1.51 (0.45-5.01) | 0.380 | 0.761 |
| Pancreas (C25) |  |  |  |  |
| No | Ref. | Ref. |  |  |
| Yes | 0.58 (0.28-1.23) | 0.58 (0.22-1.55) | 0.155 | 0.761 |
| Larynx (C32) |  |  |  |  |
| No | Ref. | Ref. |  |  |
| Yes | 1.38 (0.17-11.07) | 1.38 (0.09-21.27) | 0.760 | 0.804 |
| Other thoracic organs (C37-38) |  |  |  |  |
| No | Ref. | Ref. |  |  |
| Yes | 2.22 (0.30-16.73) | 2.22 (0.16-31.54) | 0.437 | 0.777 |
| Bone (C40-41) |  |  |  |  |
| No | Ref. | Ref. |  |  |
| Yes | 0.83 (0.19-3.60) | 0.83 (0.12-5.71) | 0.804 | 0.804 |
| Melanoma of skin (C43) |  |  |  |  |
| No | Ref. | Ref. |  |  |
| Yes | 0.53 (0.18-1.59) | 0.53 (0.13-2.25) | 0.258 | 0.761 |
| Ovary (C56) |  |  |  |  |
| No | Ref. | Ref. |  |  |
| Yes | 1.19 (0.62-2.29) | 1.19 (0.51-2.81) | 0.596 | 0.794 |
| Kidney (C64-66, C68) |  |  |  |  |
| No | Ref. | Ref. |  |  |
| Yes | 0.80 (0.40-1.62) | 0.80 (0.32-2.02) | 0.536 | 0.779 |
| Bladder (C67) |  |  |  |  |
| No | Ref. | Ref. |  |  |
| Yes | 2.17 (0.52-9.02) | 2.17 (0.33-14.11) | 0.286 | 0.761 |
| Lymphoma (C81-85, C88, C90, C96) |  |  |  |  |
| No | Ref. | Ref. |  |  |
| Yes | 0.91 (0.52-1.58) | 0.91 (0.44-1.89) | 0.732 | 0.804 |
| Leukaemia (C91-95) |  |  |  |  |
| No | Ref. | Ref. |  |  |
| Yes | 1.87 (0.68-5.13) | 1.87 (0.49-7.05) | 0.226 | 0.761 |

Multivariate-adjusted model: age at enrollment, educational level (low, medium, high), marital status (married, others), smoking status (never, current, ever), alcohol drinking status (never, ever), regular exercise (no, yes), BMI (underweight, normal, overweight, obese), family history of cancer (no, yes), hypertension (no, yes), diabetes (no, yes), age at menarche (<13, 13, 14, >14 years), and menopause status (no, yes).

**Two methods were implemented to mitigate false positives resulting from multiple comparisons:** (i) reporting 99% confidence intervals (in addition to conventional 95%CIs) to apply more stringent significance thresholds; (ii) adjusting *P*-values using the Benjamini-Hochberg false discovery rate (FDR) correction.

Abbreviations: HR, hazard ratio; CI, confidence interval; FDR, false discovery rate.

**eTable 7. Sensitivity analysis for risk of developing overall cancer and its main site-specific cancers in relationship to baseline total breastfeeding duration.**

| **Common site-specific cancers** | **Total breastfeeding duration** | **Multivariate-adjusted model** | | | |
| --- | --- | --- | --- | --- | --- |
|  |  | **HR (95%CI)** | **HR (99%CI)** | ***P*-value** | ***P*-value _FDR_** |
| Colon-rectum (C18-21) | Continuous (per 12 months) | 0.87 (0.75-1.01) | 0.87 (0.72-1.06) | 0.072 | 0.162 |
|  | Multi-categorical (month) |  |  |  |  |
|  | 0 | Ref. | Ref. |  |  |
|  | 1-6 | 1.25 (0.87-1.80) | 1.25 (0.78-2.01) |  |  |
|  | 7-12 | 1.03 (0.76-1.41) | 1.03 (0.69-1.55) |  |  |
|  | >12 | 0.92 (0.65-1.30) | 0.92 (0.58-1.44) |  |  |
|  | *P*-trend | 0.216 |  |  |  |
|  | *P*-trend _FDR_ | 0.337 |  |  |  |
| Lung (C33-34) | Continuous (per 12 months) | 0.97 (0.86-1.09) | 0.97 (0.83-1.13) | 0.577 | 0.649 |
|  | Multi-categorical (month) |  |  |  |  |
|  | 0 | Ref. | Ref. |  |  |
|  | 1-6 | 1.13 (0.82-1.54) | 1.13 (0.74-1.71) |  |  |
|  | 7-12 | 1.07 (0.82-1.39) | 1.07 (0.75-1.51) |  |  |
|  | >12 | 1.06 (0.79-1.42) | 1.06 (0.72-1.55) |  |  |
|  | *P*-trend | 0.915 |  |  |  |
|  | *P*-trend _FDR_ | 0.915 |  |  |  |
| Breast (C50) | Continuous (per 12 months) | 0.85 (0.76-0.95) | 0.85 (0.74-0.99) | 0.005 | 0.047 |
|  | Multi-categorical (month) |  |  |  |  |
|  | 0 | Ref. | Ref. |  |  |
|  | 1-6 | 0.82 (0.64-1.04) | 0.82 (0.59-1.12) |  |  |
|  | 7-12 | 0.78 (0.64-0.94) | 0.78 (0.60-1.00) |  |  |
|  | >12 | 0.75 (0.60-0.94) | 0.75 (0.56-1.01) |  |  |
|  | *P*-trend | 0.015 |  |  |  |
|  | *P*-trend _FDR_ | 0.138 |  |  |  |
| Cervix (C53) | Continuous (per 12 months) | 1.09 (0.91-1.30) | 1.09 (0.87-1.37) | 0.338 | 0.507 |
|  | Multi-categorical (month) |  |  |  |  |
|  | 0 | Ref. | Ref. |  |  |
|  | 1-6 | 1.01 (0.64-1.60) | 1.01 (0.55-1.86) |  |  |
|  | 7-12 | 1.17 (0.81-1.71) | 1.17 (0.72-1.92) |  |  |
|  | >12 | 1.30 (0.86-1.96) | 1.30 (0.75-2.24) |  |  |
|  | *P*-trend | 0.130 |  |  |  |
|  | *P*-trend _FDR_ | 0.337 |  |  |  |
| Uterus (C54-55) | Continuous (per 12 months) | 1.13 (0.90-1.41) | 1.13 (0.84-1.51) | 0.299 | 0.507 |
|  | Multi-categorical (month) |  |  |  |  |
|  | 0 | Ref. | Ref. |  |  |
|  | 1-6 | 0.73 (0.38-1.40) | 0.73 (0.31-1.72) |  |  |
|  | 7-12 | 1.23 (0.75-2.01) | 1.23 (0.64-2.35) |  |  |
|  | >12 | 1.16 (0.67-2.01) | 1.16 (0.56-2.39) |  |  |
|  | *P*-trend | 0.215 |  |  |  |
|  | *P*-trend _FDR_ | 0.337 |  |  |  |
| Brain (C70-72) | Continuous (per 12 months) | 1.00 (0.82-1.23) | 1.00 (0.77-1.31) | 0.976 | 0.976 |
|  | Multi-categorical (month) |  |  |  |  |
|  | 0 | Ref. | Ref. |  |  |
|  | 1-6 | 1.08 (0.61-1.92) | 1.08 (0.51-2.30) |  |  |
|  | 7-12 | 1.16 (0.73-1.86) | 1.16 (0.63-2.16) |  |  |
|  | >12 | 1.09 (0.65-1.84) | 1.09 (0.55-2.16) |  |  |
|  | *P*-trend | 0.743 |  |  |  |
|  | *P*-trend _FDR_ | 0.835 |  |  |  |
| Thyroid (C73) | Continuous (per 12 months) | 0.83 (0.69-0.99) | 0.83 (0.65-1.05) | 0.044 | 0.131 |
|  | Multi-categorical (month) |  |  |  |  |
|  | 0 | Ref. | Ref. |  |  |
|  | 1-6 | 1.07 (0.73-1.55) | 1.07 (0.65-1.75) |  |  |
|  | 7-12 | 0.93 (0.68-1.28) | 0.93 (0.61-1.41) |  |  |
|  | >12 | 0.78 (0.53-1.14) | 0.78 (0.47-1.28) |  |  |
|  | *P*-trend | 0.107 |  |  |  |
|  | *P*-trend _FDR_ | 0.337 |  |  |  |
| All others (NA) | Continuous (per 12 months) | 1.05 (0.89-1.24) | 1.05 (0.84-1.31) | 0.573 | 0.649 |
|  | Multi-categorical (month) |  |  |  |  |
|  | 0 | Ref. | Ref. |  |  |
|  | 1-6 | 0.99 (0.60-1.62) | 0.99 (0.52-1.89) |  |  |
|  | 7-12 | 1.25 (0.84-1.86) | 1.25 (0.74-2.11) |  |  |
|  | >12 | 1.15 (0.74-1.77) | 1.15 (0.65-2.03) |  |  |
|  | *P*-trend | 0.371 |  |  |  |
|  | *P*-trend _FDR_ | 0.477 |  |  |  |
| All sites (C00-97, D32-33, D42-43, D45-47) | Continuous (per 12 months) | 0.95 (0.90-0.99) | 0.95 (0.89-1.01) | 0.030 | 0.131 |
|  | Multi-categorical (month) |  |  |  |  |
|  | 0 | Ref. | Ref. |  |  |
|  | 1-6 | 1.02 (0.91-1.15) | 1.02 (0.87-1.19) |  |  |
|  | 7-12 | 0.99 (0.90-1.10) | 0.99 (0.88-1.13) |  |  |
|  | >12 | 0.95 (0.85-1.06) | 0.95 (0.82-1.10) |  |  |
|  | *P*-trend | 0.225 |  |  |  |
|  | *P*-trend _FDR_ | 0.337 |  |  |  |

**Two methods were implemented to mitigate false positives resulting from multiple comparisons:** (i) reporting 99% confidence intervals (in addition to conventional 95%CIs) to apply more stringent significance thresholds; (ii) adjusting *P*-values using the Benjamini-Hochberg false discovery rate (FDR) correction.

Abbreviations: HR, hazard ratio; CI, confidence interval; FDR, false discovery rate.

**eTable 8. Sensitivity analysis for risk of developing rare site-specific cancers in relationship to baseline total breastfeeding duration.**

| **Rare site-specific cancers** | **Total breastfeeding duration** | **Multivariate-adjusted model** | | | |
| --- | --- | --- | --- | --- | --- |
|  |  | **HR (95%CI)** | **HR (99%CI)** | ***P*-value** | ***P*-value _FDR_** |
| Oral cavity and pharynx (C00-10,  C12-14) | Continuous (per 12 months) | 0.70 (0.44-1.12) | 0.70 (0.38-1.29) | 0.136 | 0.724 |
|  | Multi-categorical (month) |  |  |  |  |
|  | 0 | Ref. | Ref. |  |  |
|  | 1-6 | 1.99 (0.63-6.28) | 1.99 (0.44-9.01) |  |  |
|  | 7-12 | 1.38 (0.49-3.89) | 1.38 (0.35-5.38) |  |  |
|  | >12 | 1.08 (0.34-3.40) | 1.08 (0.24-4.87) |  |  |
|  | *P*-trend | 0.590 |  |  |  |
|  | *P*-trend _FDR_ | 0.787 |  |  |  |
| Nasopharynx (C11) | Continuous (per 12 months) | 0.98 (0.61-1.59) | 0.98 (0.52-1.85) | 0.934 | 0.947 |
|  | Multi-categorical (month) |  |  |  |  |
|  | 0 | Ref. | Ref. |  |  |
|  | 1-6 | 0.60 (0.21-1.75) | 0.60 (0.15-2.45) |  |  |
|  | 7-12 | 0.49 (0.22-1.12) | 0.49 (0.17-1.45) |  |  |
|  | >12 | 0.61 (0.24-1.56) | 0.61 (0.18-2.10) |  |  |
|  | *P*-trend | 0.283 |  |  |  |
|  | *P*-trend _FDR_ | 0.648 |  |  |  |
| Oesophagus (C15) | Continuous (per 12 months) | 0.98 (0.51-1.88) | 0.98 (0.42-2.30) | 0.947 | 0.947 |
|  | Multi-categorical (month) |  |  |  |  |
|  | 0 | Ref. | Ref. |  |  |
|  | 1-6 | 2.38 (0.25-23.04) | 2.38 (0.12-47.05) |  |  |
|  | 7-12 | 2.96 (0.39-22.49) | 2.96 (0.21-42.55) |  |  |
|  | >12 | 1.62 (0.18-14.81) | 1.62 (0.09-29.70) |  |  |
|  | *P*-trend | 0.834 |  |  |  |
|  | *P*-trend _FDR_ | 0.889 |  |  |  |
| Stomach (C16) | Continuous (per 12 months) | 1.03 (0.80-1.34) | 1.03 (0.74-1.45) | 0.798 | 0.947 |
|  | Multi-categorical (month) |  |  |  |  |
|  | 0 | Ref. | Ref. |  |  |
|  | 1-6 | 1.44 (0.66-3.14) | 1.44 (0.52-4.01) |  |  |
|  | 7-12 | 1.57 (0.81-3.04) | 1.57 (0.66-3.74) |  |  |
|  | >12 | 1.37 (0.67-2.82) | 1.37 (0.53-3.53) |  |  |
|  | *P*-trend | 0.497 |  |  |  |
|  | *P*-trend _FDR_ | 0.787 |  |  |  |
| Liver (C22) | Continuous (per 12 months) | 0.79 (0.61-1.03) | 0.79 (0.57-1.11) | 0.080 | 0.642 |
|  | Multi-categorical (month) |  |  |  |  |
|  | 0 | Ref. | Ref. |  |  |
|  | 1-6 | 1.32 (0.73-2.38) | 1.32 (0.61-2.86) |  |  |
|  | 7-12 | 0.81 (0.49-1.35) | 0.81 (0.42-1.59) |  |  |
|  | >12 | 0.86 (0.49-1.51) | 0.86 (0.41-1.81) |  |  |
|  | *P*-trend | 0.192 |  |  |  |
|  | *P*-trend _FDR_ | 0.648 |  |  |  |
| Gallbladder (C23-24) | Continuous (per 12 months) | 1.51 (1.12-2.02) | 1.51 (1.02-2.22) | 0.007 | 0.106 |
|  | Multi-categorical (month) |  |  |  |  |
|  | 0 | Ref. | Ref. |  |  |
|  | 1-6 | 1.13 (0.36-3.59) | 1.13 (0.25-5.15) |  |  |
|  | 7-12 | 1.33 (0.52-3.41) | 1.33 (0.39-4.58) |  |  |
|  | >12 | 2.19 (0.83-5.76) | 2.19 (0.61-7.81) |  |  |
|  | *P*-trend | 0.039 |  |  |  |
|  | *P*-trend _FDR_ | 0.630 |  |  |  |
| Pancreas (C25) | Continuous (per 12 months) | 0.94 (0.63-1.41) | 0.94 (0.56-1.59) | 0.776 | 0.947 |
|  | Multi-categorical (month) |  |  |  |  |
|  | 0 | Ref. | Ref. |  |  |
|  | 1-6 | 0.74 (0.28-1.99) | 0.74 (0.20-2.72) |  |  |
|  | 7-12 | 0.58 (0.27-1.25) | 0.58 (0.21-1.59) |  |  |
|  | >12 | 0.52 (0.22-1.25) | 0.52 (0.17-1.65) |  |  |
|  | *P*-trend | 0.129 |  |  |  |
|  | *P*-trend _FDR_ | 0.648 |  |  |  |
| Larynx (C32) | Continuous (per 12 months) | 0.75 (0.29-1.92) | 0.75 (0.22-2.57) | 0.549 | 0.878 |
|  | Multi-categorical (month) |  |  |  |  |
|  | 0 | Ref. | Ref. |  |  |
|  | 1-6 | 2.87 (0.29-28.54) | 2.87 (0.14-58.71) |  |  |
|  | 7-12 | 1.02 (0.12-8.85) | 1.02 (0.06-17.44) |  |  |
|  | >12 | 1.54 (0.16-14.61) | 1.54 (0.08-29.64) |  |  |
|  | *P*-trend | 0.898 |  |  |  |
|  | *P*-trend _FDR_ | 0.898 |  |  |  |
| Other thoracic organs (C37-38) | Continuous (per 12 months) | 1.15 (0.58-2.30) | 1.15 (0.46-2.86) | 0.686 | 0.947 |
|  | Multi-categorical (month) |  |  |  |  |
|  | 0 | Ref. | Ref. |  |  |
|  | 1-6 | 2.70 (0.30-24.37) | 2.70 (0.15-48.64) |  |  |
|  | 7-12 | 1.96 (0.25-15.23) | 1.96 (0.13-29.02) |  |  |
|  | >12 | 2.64 (0.31-22.55) | 2.64 (0.16-44.24) |  |  |
|  | *P*-trend | 0.530 |  |  |  |
|  | *P*-trend _FDR_ | 0.787 |  |  |  |
| Bone (C40-41) | Continuous (per 12 months) | 1.32 (0.72-2.42) | 1.32 (0.59-2.93) | 0.371 | 0.861 |
|  | Multi-categorical (month) |  |  |  |  |
|  | 0 | Ref. | Ref. |  |  |
|  | 1-6 | 1.01 (0.17-6.07) | 1.01 (0.10-10.67) |  |  |
|  | 7-12 | 0.63 (0.14-2.95) | 0.63 (0.08-4.78) |  |  |
|  | >12 | 1.24 (0.25-6.04) | 1.24 (0.15-9.94) |  |  |
|  | *P*-trend | 0.731 |  |  |  |
|  | *P*-trend _FDR_ | 0.889 |  |  |  |
| Melanoma of skin (C43) | Continuous (per 12 months) | 0.72 (0.34-1.52) | 0.72 (0.27-1.93) | 0.393 | 0.861 |
|  | Multi-categorical (month) |  |  |  |  |
|  | 0 | Ref. | Ref. |  |  |
|  | 1-6 | 0.78 (0.19-3.16) | 0.78 (0.12-4.91) |  |  |
|  | 7-12 | 0.47 (0.15-1.52) | 0.47 (0.10-2.18) |  |  |
|  | >12 | 0.52 (0.13-2.03) | 0.52 (0.09-3.11) |  |  |
|  | *P*-trend | 0.248 |  |  |  |
|  | *P*-trend _FDR_ | 0.648 |  |  |  |
| Ovary (C56) | Continuous (per 12 months) | 0.83 (0.60-1.15) | 0.83 (0.54-1.27) | 0.259 | 0.861 |
|  | Multi-categorical (month) |  |  |  |  |
|  | 0 | Ref. | Ref. |  |  |
|  | 1-6 | 1.50 (0.70-3.19) | 1.50 (0.55-4.04) |  |  |
|  | 7-12 | 1.26 (0.65-2.44) | 1.26 (0.53-3.01) |  |  |
|  | >12 | 0.75 (0.34-1.66) | 0.75 (0.26-2.13) |  |  |
|  | *P*-trend | 0.273 |  |  |  |
|  | *P*-trend _FDR_ | 0.648 |  |  |  |
| Kidney (C64-66, C68) | Continuous (per 12 months) | 1.03 (0.72-1.47) | 1.03 (0.64-1.64) | 0.878 | 0.947 |
|  | Multi-categorical (month) |  |  |  |  |
|  | 0 | Ref. | Ref. |  |  |
|  | 1-6 | 0.34 (0.11-1.12) | 0.34 (0.07-1.62) |  |  |
|  | 7-12 | 0.90 (0.44-1.84) | 0.90 (0.35-2.31) |  |  |
|  | >12 | 0.81 (0.36-1.82) | 0.81 (0.28-2.35) |  |  |
|  | *P*-trend | 0.797 |  |  |  |
|  | *P*-trend _FDR_ | 0.889 |  |  |  |
| Bladder (C67) | Continuous (per 12 months) | 1.18 (0.76-1.83) | 1.18 (0.66-2.10) | 0.468 | 0.861 |
|  | Multi-categorical (month) |  |  |  |  |
|  | 0 | Ref. | Ref. |  |  |
|  | 1-6 | 2.17 (0.44-10.79) | 2.17 (0.26-17.85) |  |  |
|  | 7-12 | 2.02 (0.48-8.57) | 2.02 (0.30-13.49) |  |  |
|  | >12 | 2.59 (0.58-11.60) | 2.59 (0.36-18.57) |  |  |
|  | *P*-trend | 0.248 |  |  |  |
|  | *P*-trend _FDR_ | 0.648 |  |  |  |
| Lymphoma (C81-85, C88, C90, C96) | Continuous (per 12 months) | 0.90 (0.68-1.20) | 0.90 (0.62-1.31) | 0.478 | 0.861 |
|  | Multi-categorical (month) |  |  |  |  |
|  | 0 | Ref. | Ref. |  |  |
|  | 1-6 | 0.98 (0.48-1.97) | 0.98 (0.39-2.46) |  |  |
|  | 7-12 | 0.91 (0.52-1.61) | 0.91 (0.43-1.93) |  |  |
|  | >12 | 0.86 (0.45-1.62) | 0.86 (0.37-1.98) |  |  |
|  | *P*-trend | 0.575 |  |  |  |
|  | *P*-trend _FDR_ | 0.787 |  |  |  |
| Leukaemia (C91-95) | Continuous (per 12 months) | 1.13 (0.80-1.61) | 1.13 (0.72-1.79) | 0.484 | 0.861 |
|  | Multi-categorical (month) |  |  |  |  |
|  | 0 | Ref. | Ref. |  |  |
|  | 1-6 | 1.61 (0.50-5.26) | 1.61 (0.34-7.62) |  |  |
|  | 7-12 | 2.00 (0.72-5.57) | 2.00 (0.52-7.67) |  |  |
|  | >12 | 1.65 (0.55-4.97) | 1.65 (0.39-7.02) |  |  |
|  | *P*-trend | 0.440 |  |  |  |
|  | *P*-trend _FDR_ | 0.787 |  |  |  |

**Two methods were implemented to mitigate false positives resulting from multiple comparisons:** (i) reporting 99% confidence intervals (in addition to conventional 95%CIs) to apply more stringent significance thresholds; (ii) adjusting *P*-values using the Benjamini-Hochberg false discovery rate (FDR) correction.

Abbreviations: HR, hazard ratio; CI, confidence interval; FDR, false discovery rate.

**eTable 9. Sensitivity analysis for further adjusting for surgeries on the reproductive system among parous women (2012-2019).**

| Site-specific cancers | History of breastfeeding | |  | Total breastfeeding duration | | |
| --- | --- | --- | --- | --- | --- | --- |
|  |  |  |  | Multi-categorical (month) | | Continuous  (per 12 months) |
|  |  | HR (95%CI) |  |  | HR (95%CI) | HR (95%CI) |
| Oral cavity and pharynx (C00-10, C12-14) |  |  |  |  |  |  |
|  | No | Ref. |  | 0 | Ref. |  |
|  | Yes | 2.68 (0.65-11.05) |  | 1-6 | 3.57 (0.78-16.36) | 0.80 (0.50-1.26) |
|  |  |  |  | 7-12 | 2.69 (0.65-11.23) |  |
|  |  |  |  | >12 | 2.13 (0.47-9.64) |  |
| Nasopharynx (C11) |  |  |  |  |  |  |
|  | No | Ref. |  | 0 | Ref. |  |
|  | Yes | 0.62 (0.26-1.50) |  | 1-6 | 0.75 (0.24-2.34) | 1.13 (0.69-1.84) |
|  |  |  |  | 7-12 | 0.53 (0.21-1.35) |  |
|  |  |  |  | >12 | 0.78 (0.28-2.17) |  |
| Oesophagus (C15) |  |  |  |  |  |  |
|  | No | Ref. |  | 0 | Ref. |  |
|  | Yes | 2.26 (0.30-16.99) |  | 1-6 | 2.21 (0.23-21.44) | 0.99 (0.50-1.96) |
|  |  |  |  | 7-12 | 2.52 (0.33-19.26) |  |
|  |  |  |  | >12 | 1.56 (0.17-14.29) |  |
| Stomach (C16) |  |  |  |  |  |  |
|  | No | Ref. |  | 0 | Ref. |  |
|  | Yes | 1.66 (0.81-3.41) |  | 1-6 | 1.42 (0.60-3.36) | 1.10 (0.84-1.44) |
|  |  |  |  | 7-12 | 1.72 (0.83-3.58) |  |
|  |  |  |  | >12 | 1.63 (0.74-3.57) |  |
| Colon-rectum (C18-21) |  |  |  |  |  |  |
|  | No | Ref. |  | 0 | Ref. |  |
|  | Yes | 1.03 (0.75-1.41) |  | 1-6 | 1.24 (0.85-1.80) | 0.89 (0.76-1.03) |
|  |  |  |  | 7-12 | 1.01 (0.73-1.40) |  |
|  |  |  |  | >12 | 0.93 (0.65-1.33) |  |
| Liver (C22) |  |  |  |  |  |  |
|  | No | Ref. |  | 0 | Ref. |  |
|  | Yes | 0.92 (0.55-1.52) |  | 1-6 | 1.38 (0.76-2.52) | 0.81 (0.62-1.05) |
|  |  |  |  | 7-12 | 0.83 (0.49-1.41) |  |
|  |  |  |  | >12 | 0.87 (0.48-1.55) |  |
| Gallbladder (C23-24) |  |  |  |  |  |  |
|  | No | Ref. |  | 0 | Ref. |  |
|  | Yes | 1.62 (0.59-4.48) |  | 1-6 | 1.15 (0.32-4.08) | 1.66 (1.22-2.25) |
|  |  |  |  | 7-12 | 1.37 (0.48-3.87) |  |
|  |  |  |  | >12 | 2.60 (0.90-7.54) |  |
| Pancreas (C25) |  |  |  |  |  |  |
|  | No | Ref. |  | 0 | Ref. |  |
|  | Yes | 0.58 (0.27-1.22) |  | 1-6 | 0.74 (0.28-1.99) | 0.88 (0.58-1.33) |
|  |  |  |  | 7-12 | 0.58 (0.27-1.27) |  |
|  |  |  |  | >12 | 0.50 (0.21-1.21) |  |
| Larynx (C32) |  |  |  |  |  |  |
|  | No | Ref. |  | 0 | Ref. |  |
|  | Yes | 1.43 (0.18-11.70) |  | 1-6 | 2.90 (0.29-29.19) | 0.76 (0.30-1.94) |
|  |  |  |  | 7-12 | 1.05 (0.12-9.28) |  |
|  |  |  |  | >12 | 1.60 (0.17-15.34) |  |
| Lung (C33-34) |  |  |  |  |  |  |
|  | No | Ref. |  | 0 | Ref. |  |
|  | Yes | 1.08 (0.83-1.41) |  | 1-6 | 1.15 (0.83-1.59) | 0.95 (0.84-1.07) |
|  |  |  |  | 7-12 | 1.08 (0.82-1.42) |  |
|  |  |  |  | >12 | 1.04 (0.77-1.41) |  |
| Other thoracic organs (C37-38) |  |  |  |  |  |  |
|  | No | Ref. |  | 0 | Ref. |  |
|  | Yes | 2.08 (0.28-15.74) |  | 1-6 | 2.59 (0.29-23.35) | 1.06 (0.50-2.26) |
|  |  |  |  | 7-12 | 1.88 (0.24-14.67) |  |
|  |  |  |  | >12 | 2.28 (0.26-20.10) |  |
| Bone (C40-41) |  |  |  |  |  |  |
|  | No | Ref. |  | 0 | Ref. |  |
|  | Yes | 0.83 (0.19-3.62) |  | 1-6 | 1.00 (0.17-6.01) | 1.32 (0.72-2.43) |
|  |  |  |  | 7-12 | 0.63 (0.14-2.95) |  |
|  |  |  |  | >12 | 1.25 (0.26-6.07) |  |
| Melanoma of skin (C43) |  |  |  |  |  |  |
|  | No | Ref. |  | 0 | Ref. |  |
|  | Yes | 0.50 (0.17-1.50) |  | 1-6 | 0.75 (0.18-3.04) | 0.72 (0.34-1.55) |
|  |  |  |  | 7-12 | 0.44 (0.14-1.40) |  |
|  |  |  |  | >12 | 0.51 (0.13-1.99) |  |
| Breast (C50) |  |  |  |  |  |  |
|  | No | Ref. |  | 0 | Ref. |  |
|  | Yes | 0.84 (0.68-1.02) |  | 1-6 | 0.88 (0.68-1.14) | 0.88 (0.79-0.99) |
|  |  |  |  | 7-12 | 0.83 (0.67-1.02) |  |
|  |  |  |  | >12 | 0.83 (0.65-1.06) |  |
| Cervix (C53) |  |  |  |  |  |  |
|  | No | Ref. |  | 0 | Ref. |  |
|  | Yes | 1.11 (0.77-1.62) |  | 1-6 | 0.95 (0.59-1.53) | 1.06 (0.88-1.27) |
|  |  |  |  | 7-12 | 1.13 (0.77-1.65) |  |
|  |  |  |  | >12 | 1.20 (0.78-1.83) |  |
| Uterus (C54-55) |  |  |  |  |  |  |
|  | No | Ref. |  | 0 | Ref. |  |
|  | Yes | 1.10 (0.66-1.81) |  | 1-6 | 0.74 (0.38-1.44) | 1.11 (0.88-1.41) |
|  |  |  |  | 7-12 | 1.18 (0.71-1.97) |  |
|  |  |  |  | >12 | 1.08 (0.61-1.92) |  |
| Ovary (C56) |  |  |  |  |  |  |
|  | No | Ref. |  | 0 | Ref. |  |
|  | Yes | 1.29 (0.65-2.56) |  | 1-6 | 1.55 (0.70-3.41) | 0.84 (0.61-1.17) |
|  |  |  |  | 7-12 | 1.38 (0.69-2.78) |  |
|  |  |  |  | >12 | 0.79 (0.34-1.81) |  |
| Kidney (C64-66, C68) |  |  |  |  |  |  |
|  | No | Ref. |  | 0 | Ref. |  |
|  | Yes | 0.84 (0.40-1.76) |  | 1-6 | 0.28 (0.08-1.08) | 1.08 (0.75-1.56) |
|  |  |  |  | 7-12 | 0.96 (0.45-2.05) |  |
|  |  |  |  | >12 | 0.81 (0.34-1.92) |  |
| Bladder (C67) |  |  |  |  |  |  |
|  | No | Ref. |  | 0 | Ref. |  |
|  | Yes | 2.10 (0.51-8.73) |  | 1-6 | 2.10 (0.42-10.46) | 1.25 (0.80-1.95) |
|  |  |  |  | 7-12 | 1.90 (0.45-8.07) |  |
|  |  |  |  | >12 | 2.68 (0.60-11.99) |  |
| Brain (C70-72) |  |  |  |  |  |  |
|  | No | Ref. |  | 0 | Ref. |  |
|  | Yes | 1.15 (0.71-1.84) |  | 1-6 | 1.03 (0.57-1.86) | 1.05 (0.86-1.29) |
|  |  |  |  | 7-12 | 1.16 (0.72-1.88) |  |
|  |  |  |  | >12 | 1.18 (0.70-2.00) |  |
| Thyroid (C73) |  |  |  |  |  |  |
|  | No | Ref. |  | 0 | Ref. |  |
|  | Yes | 0.97 (0.70-1.33) |  | 1-6 | 1.13 (0.77-1.67) | 0.82 (0.68-0.99) |
|  |  |  |  | 7-12 | 0.98 (0.70-1.36) |  |
|  |  |  |  | >12 | 0.78 (0.52-1.16) |  |
| Lymphoma (C81-85, C88, C90, C96) |  |  |  |  |  |  |
|  | No | Ref. |  | 0 | Ref. |  |
|  | Yes | 0.90 (0.50-1.60) |  | 1-6 | 1.01 (0.49-2.07) | 0.88 (0.65-1.19) |
|  |  |  |  | 7-12 | 0.91 (0.50-1.65) |  |
|  |  |  |  | >12 | 0.80 (0.41-1.55) |  |
| Leukaemia (C91-95) |  |  |  |  |  |  |
|  | No | Ref. |  | 0 | Ref. |  |
|  | Yes | 2.34 (0.73-7.46) |  | 1-6 | 1.85 (0.49-6.97) | 1.23 (0.86-1.75) |
|  |  |  |  | 7-12 | 2.50 (0.77-8.05) |  |
|  |  |  |  | >12 | 2.23 (0.64-7.69) |  |
| All others (NA) |  |  |  |  |  |  |
|  | No | Ref. |  | 0 | Ref. |  |
|  | Yes | 1.16 (0.78-1.72) |  | 1-6 | 0.99 (0.60-1.63) | 1.05 (0.88-1.25) |
|  |  |  |  | 7-12 | 1.21 (0.81-1.81) |  |
|  |  |  |  | >12 | 1.12 (0.72-1.75) |  |
| All sites (C00-97, D32-33, D42-43, D45-47) |  |  |  |  |  |  |
|  | No | Ref. |  | 0 | Ref. |  |
|  | Yes | 1.02 (0.92-1.12) |  | 1-6 | 1.05 (0.93-1.19) | 0.96 (0.91-1.01) |
|  |  |  |  | 7-12 | 1.02 (0.92-1.13) |  |
|  |  |  |  | >12 | 0.98 (0.88-1.10) |  |

The Cox regression model adjusted for age at enrollment (continuous), educational level (low, medium, high), marital status (married, others), smoking status (never, current, ever), alcohol drinking status (never, ever), regular exercise (no, yes), BMI (underweight, normal, overweight, obese), family history of cancer (no, yes), hypertension (no, yes), diabetes (no, yes), and age at menarche (<13, 13, 14, >14 years), menopause status (no, yes), and surgeries on the reproductive system (no, yes).

Abbreviations: HR, hazard ratio; CI, confidence interval.

**eTable 10. Sensitivity analysis for including nulliparous female participants.**

| Site-specific cancers | History of breastfeeding | |  | Total breastfeeding duration | | |
| --- | --- | --- | --- | --- | --- | --- |
|  |  |  |  | Multi-categorical (month) | | Continuous  (per 12 months) |
|  |  | HR (95%CI) |  |  | HR (95%CI) | HR (95%CI) |
| Oral cavity and pharynx (C00-10, C12-14) |  |  |  |  |  |  |
|  | No | Ref. |  | 0 | Ref. |  |
|  | Yes | 1.47 (0.59-3.70) |  | 1-6 | 2.11 (0.73-6.11) | 0.74 (0.48-1.14) |
|  |  |  |  | 7-12 | 1.47 (0.57-3.75) |  |
|  |  |  |  | >12 | 1.13 (0.39-3.25) |  |
| Nasopharynx (C11) |  |  |  |  |  |  |
|  | No | Ref. |  | 0 | Ref. |  |
|  | Yes | 0.45 (0.23-0.87) |  | 1-6 | 0.51 (0.19-1.38) | 0.87 (0.54-1.40) |
|  |  |  |  | 7-12 | 0.41 (0.20-0.83) |  |
|  |  |  |  | >12 | 0.52 (0.22-1.21) |  |
| Oesophagus (C15) |  |  |  |  |  |  |
|  | No | Ref. |  | 0 | Ref. |  |
|  | Yes | 3.13 (0.42-23.38) |  | 1-6 | 2.90 (0.30-28.08) | 1.03 (0.55-1.94) |
|  |  |  |  | 7-12 | 3.62 (0.48-27.39) |  |
|  |  |  |  | >12 | 1.98 (0.22-18.01) |  |
| Stomach (C16) |  |  |  |  |  |  |
|  | No | Ref. |  | 0 | Ref. |  |
|  | Yes | 1.56 (0.86-2.82) |  | 1-6 | 1.49 (0.72-3.10) | 1.06 (0.83-1.37) |
|  |  |  |  | 7-12 | 1.62 (0.89-2.97) |  |
|  |  |  |  | >12 | 1.42 (0.73-2.78) |  |
| Colon-rectum (C18-21) |  |  |  |  |  |  |
|  | No | Ref. |  | 0 | Ref. |  |
|  | Yes | 1.05 (0.80-1.37) |  | 1-6 | 1.26 (0.90-1.77) | 0.89 (0.77-1.02) |
|  |  |  |  | 7-12 | 1.05 (0.79-1.38) |  |
|  |  |  |  | >12 | 0.93 (0.67-1.28) |  |
| Liver (C22) |  |  |  |  |  |  |
|  | No | Ref. |  | 0 | Ref. |  |
|  | Yes | 1.03 (0.65-1.65) |  | 1-6 | 1.53 (0.87-2.70) | 0.84 (0.66-1.08) |
|  |  |  |  | 7-12 | 0.94 (0.58-1.53) |  |
|  |  |  |  | >12 | 1.01 (0.59-1.73) |  |
| Gallbladder (C23-24) |  |  |  |  |  |  |
|  | No | Ref. |  | 0 | Ref. |  |
|  | Yes | 1.15 (0.55-2.41) |  | 1-6 | 0.87 (0.31-2.40) | 1.43 (1.07-1.91) |
|  |  |  |  | 7-12 | 1.02 (0.47-2.19) |  |
|  |  |  |  | >12 | 1.66 (0.74-3.70) |  |
| Pancreas (C25) |  |  |  |  |  |  |
|  | No | Ref. |  | 0 | Ref. |  |
|  | Yes | 0.61 (0.31-1.21) |  | 1-6 | 0.78 (0.30-1.99) | 0.93 (0.63-1.36) |
|  |  |  |  | 7-12 | 0.61 (0.30-1.24) |  |
|  |  |  |  | >12 | 0.54 (0.24-1.23) |  |
| Larynx (C32) |  |  |  |  |  |  |
|  | No | Ref. |  | 0 | Ref. |  |
|  | Yes | 1.62 (0.21-12.74) |  | 1-6 | 3.34 (0.34-32.67) | 0.80 (0.32-1.97) |
|  |  |  |  | 7-12 | 1.20 (0.14-10.25) |  |
|  |  |  |  | >12 | 1.81 (0.20-16.82) |  |
| Lung (C33-34) |  |  |  |  |  |  |
|  | No | Ref. |  | 0 | Ref. |  |
|  | Yes | 0.95 (0.77-1.19) |  | 1-6 | 1.00 (0.75-1.33) | 0.94 (0.84-1.06) |
|  |  |  |  | 7-12 | 0.95 (0.76-1.19) |  |
|  |  |  |  | >12 | 0.94 (0.73-1.21) |  |
| Other thoracic organs (C37-38) |  |  |  |  |  |  |
|  | No | Ref. |  | 0 | Ref. |  |
|  | Yes | 1.48 (0.34-6.43) |  | 1-6 | 1.87 (0.34-10.35) | 1.09 (0.56-2.15) |
|  |  |  |  | 7-12 | 1.30 (0.29-5.89) |  |
|  |  |  |  | >12 | 1.74 (0.34-8.91) |  |
| Bone (C40-41) |  |  |  |  |  |  |
|  | No | Ref. |  | 0 | Ref. |  |
|  | Yes | 0.52 (0.17-1.53) |  | 1-6 | 0.64 (0.14-2.90) | 1.15 (0.62-2.13) |
|  |  |  |  | 7-12 | 0.39 (0.12-1.26) |  |
|  |  |  |  | >12 | 0.78 (0.23-2.68) |  |
| Melanoma of skin (C43) |  |  |  |  |  |  |
|  | No | Ref. |  | 0 | Ref. |  |
|  | Yes | 0.51 (0.19-1.39) |  | 1-6 | 0.77 (0.21-2.91) | 0.69 (0.33-1.42) |
|  |  |  |  | 7-12 | 0.46 (0.16-1.34) |  |
|  |  |  |  | >12 | 0.50 (0.14-1.78) |  |
| Breast (C50) |  |  |  |  |  |  |
|  | No | Ref. |  | 0 | Ref. |  |
|  | Yes | 0.76 (0.64-0.90) |  | 1-6 | 0.79 (0.63-1.00) | 0.84 (0.75-0.93) |
|  |  |  |  | 7-12 | 0.76 (0.64-0.90) |  |
|  |  |  |  | >12 | 0.73 (0.59-0.90) |  |
| Cervix (C53) |  |  |  |  |  |  |
|  | No | Ref. |  | 0 | Ref. |  |
|  | Yes | 1.15 (0.83-1.60) |  | 1-6 | 0.99 (0.64-1.53) | 1.08 (0.91-1.28) |
|  |  |  |  | 7-12 | 1.16 (0.83-1.62) |  |
|  |  |  |  | >12 | 1.26 (0.86-1.84) |  |
| Uterus (C54-55) |  |  |  |  |  |  |
|  | No | Ref. |  | 0 | Ref. |  |
|  | Yes | 1.06 (0.69-1.63) |  | 1-6 | 0.68 (0.37-1.25) | 1.10 (0.89-1.37) |
|  |  |  |  | 7-12 | 1.15 (0.74-1.77) |  |
|  |  |  |  | >12 | 1.07 (0.65-1.77) |  |
| Ovary (C56) |  |  |  |  |  |  |
|  | No | Ref. |  | 0 | Ref. |  |
|  | Yes | 1.23 (0.68-2.25) |  | 1-6 | 1.56 (0.76-3.17) | 0.85 (0.62-1.17) |
|  |  |  |  | 7-12 | 1.30 (0.70-2.39) |  |
|  |  |  |  | >12 | 0.78 (0.37-1.65) |  |
| Kidney (C64-66, C68) |  |  |  |  |  |  |
|  | No | Ref. |  | 0 | Ref. |  |
|  | Yes | 0.90 (0.46-1.76) |  | 1-6 | 0.39 (0.12-1.24) | 1.04 (0.74-1.47) |
|  |  |  |  | 7-12 | 1.01 (0.51-2.01) |  |
|  |  |  |  | >12 | 0.90 (0.41-1.98) |  |
| Bladder (C67) |  |  |  |  |  |  |
|  | No | Ref. |  | 0 | Ref. |  |
|  | Yes | 1.15 (0.45-2.94) |  | 1-6 | 1.17 (0.36-3.87) | 1.06 (0.69-1.64) |
|  |  |  |  | 7-12 | 1.08 (0.41-2.83) |  |
|  |  |  |  | >12 | 1.35 (0.47-3.83) |  |
| Brain (C70-72) |  |  |  |  |  |  |
|  | No | Ref. |  | 0 | Ref. |  |
|  | Yes | 1.11 (0.74-1.67) |  | 1-6 | 1.06 (0.62-1.80) | 1.00 (0.82-1.22) |
|  |  |  |  | 7-12 | 1.14 (0.75-1.73) |  |
|  |  |  |  | >12 | 1.07 (0.67-1.71) |  |
| Thyroid (C73) |  |  |  |  |  |  |
|  | No | Ref. |  | 0 | Ref. |  |
|  | Yes | 0.87 (0.66-1.14) |  | 1-6 | 1.01 (0.71-1.42) | 0.81 (0.68-0.97) |
|  |  |  |  | 7-12 | 0.87 (0.66-1.16) |  |
|  |  |  |  | >12 | 0.73 (0.52-1.04) |  |
| Lymphoma (C81-85, C88, C90, C96) |  |  |  |  |  |  |
|  | No | Ref. |  | 0 | Ref. |  |
|  | Yes | 1.02 (0.60-1.72) |  | 1-6 | 1.09 (0.55-2.14) | 0.92 (0.70-1.21) |
|  |  |  |  | 7-12 | 1.03 (0.60-1.77) |  |
|  |  |  |  | >12 | 0.95 (0.52-1.75) |  |
| Leukaemia (C91-95) |  |  |  |  |  |  |
|  | No | Ref. |  | 0 | Ref. |  |
|  | Yes | 1.41 (0.64-3.07) |  | 1-6 | 1.22 (0.45-3.28) | 1.12 (0.79-1.57) |
|  |  |  |  | 7-12 | 1.50 (0.68-3.32) |  |
|  |  |  |  | >12 | 1.26 (0.51-3.08) |  |
| All others (NA) |  |  |  |  |  |  |
|  | No | Ref. |  | 0 | Ref. |  |
|  | Yes | 1.15 (0.82-1.61) |  | 1-6 | 0.96 (0.61-1.51) | 1.05 (0.89-1.23) |
|  |  |  |  | 7-12 | 1.21 (0.85-1.71) |  |
|  |  |  |  | >12 | 1.11 (0.75-1.64) |  |
| All sites (C00-97, D32-33, D42-43, D45-47) |  |  |  |  |  |  |
|  | No | Ref. |  | 0 | Ref. |  |
|  | Yes | 0.96 (0.88-1.04) |  | 1-6 | 0.99 (0.89-1.10) | 0.94 (0.90-0.98) |
|  |  |  |  | 7-12 | 0.96 (0.88-1.05) |  |
|  |  |  |  | >12 | 0.92 (0.83-1.01) |  |

The Cox regression model adjusted for age at enrollment (continuous), educational level (low, medium, high), marital status (married, others), smoking status (never, current, ever), alcohol drinking status (never, ever), regular exercise (no, yes), BMI (underweight, normal, overweight, obese), family history of cancer (no, yes), hypertension (no, yes), diabetes (no, yes), and age at menarche (<13, 13, 14, >14 years), and menopause status (no, yes).

For nulliparous female participants, the history of breastfeeding and total breastfeeding duration were considered as none/zero.

Abbreviations: HR, hazard ratio; CI, confidence interval.
